# X-linked primary ciliary dyskinesia due to mutations in the cytoplasmic axonemal dynein assembly factor PIH1D3

**DOI:** 10.1038/ncomms14279

**Published:** 2017-02-08

**Authors:** Chiara Olcese, Mitali P. Patel, Amelia Shoemark, Santeri Kiviluoto, Marie Legendre, Hywel J. Williams, Cara K. Vaughan, Jane Hayward, Alice Goldenberg, Richard D. Emes, Mustafa M. Munye, Laura Dyer, Thomas Cahill, Jeremy Bevillard, Corinne Gehrig, Michel Guipponi, Sandra Chantot, Philippe Duquesnoy, Lucie Thomas, Ludovic Jeanson, Bruno Copin, Aline Tamalet, Christel Thauvin-Robinet, Jean- François Papon, Antoine Garin, Isabelle Pin, Gabriella Vera, Paul Aurora, Mahmoud R. Fassad, Lucy Jenkins, Christopher Boustred, Thomas Cullup, Mellisa Dixon, Alexandros Onoufriadis, Andrew Bush, Eddie M. K. Chung, Stylianos E. Antonarakis, Michael R. Loebinger, Robert Wilson, Miguel Armengot, Estelle Escudier, Claire Hogg, UK10K Rare Group, Saeed Al-Turki, Carl Anderson, Dinu Antony, Inês Barroso, Philip L. Beales, Jamie Bentham, Shoumo Bhattacharya, Keren Carss, Krishna Chatterjee, Sebahattin Cirak, Catherine Cosgrove, Daly Allan, Richard Durbin, David Fitzpatrick, Jamie Floyd, A. Reghan Foley, Chris Franklin, Marta Futema, Steve E. Humphries, Matt Hurles, Shane McCarthy, Dawn Muddyman, Francesco Muntoni, Victoria Parker, Felicity Payne, Vincent Plagnol, Lucy Raymond, David B. Savage, Peter J. Scambler, Miriam Schmidts, Robert Semple, Eva Serra, Jim Stalker, Margriet van Kogelenberg, Parthiban Vijayarangakannan, Klaudia Walter, Serge Amselem, Zhaoxia Sun, Lucia Bartoloni, Jean-Louis Blouin, Hannah M. Mitchison

**Affiliations:** 1 Department of Genetic Medicine and Development, University of Geneva School of Medicine, CH-1211 Geneva, Switzerland; 2 Department of Life Sciences and Biotechnologies, University of Ferrara, 46-44121 Ferrara, Italy; 3 Genetics and Genomic Medicine, University College London (UCL) Great Ormond Street Institute of Child Health, Guilford Street, London WC1N 1EH, UK; 4 Paediatric Department, Royal Brompton Hospital, Sydney Street, London SW3 6NP, UK; 5 Department of Genetics, Yale University School of Medicine, 333 Cedar Street, New Haven, Connecticut 06520, USA; 6 Sorbonne Universités, UPMC Univ Paris 06, INSERM UMR_S933 and Service de Génétique et Embryologie Médicales, Hôpital Armand-Trousseau, AP-HP, Paris 75012, France; 7 GOSgene, Genetics and Genomic Medicine Programme, University College London (UCL) Great Ormond Street Institute of Child Health, 30 Guilford Street, London WC1N 1EH, UK; 8 Institute of Structural and Molecular Biology, University College London and Birkbeck College, Biological Sciences, Malet Street, London, WC1E 7HX, UK; 9 Service de Génétique, CHU de Rouen, INSERM U1079, Université de Rouen, Centre Normand de Génomique Médicale et Médecine Personnalisée, Rouen, France; 10 School of Veterinary Medicine and Science, University of Nottingham, Sutton Bonington Campus, Leicestershire LE12 5RD, UK; 11 Advanced Data Analysis Centre, University of Nottingham, Sutton Bonington Campus, Leicestershire LE12 5RD, UK; 12 Department of Genetic Medicine and Laboratory, University Hospitals of Geneva, CH-1211 Geneva, Switzerland; 13 Service de Pneumologie Pédiatrique, Centre National de Référence des Maladies Respiratoires Rares, Hôpital Armand-Trousseau, AP-HP, Paris 75012, France; 14 Centre de génétique, CHU Dijon Bourgogne, Équipe EA4271 GAD, Université de Bourgogne, Hôpital François Mitterrand, 21000 Dijon, France; 15 Service d'Oto-Rhino-Laryngologie et de Chirurgie Cervico-Maxillo-Faciale, Hôpital Bicêtre, AP-HP, Le Kremlin-Bicêtre 94275, France; 16 Pédiatrie, CHU Grenoble Alpes, INSERM U 1209, Institut for Advanced Biosciences, Université Grenoble Alpes, Grenoble, France; 17 Department of Paediatric Respiratory Medicine, Great Ormond Street Hospital for Children, London WC1N 3JH, UK; 18 Department of Respiratory, Critical Care and Anaesthesia Unit, University College London (UCL) Great Ormond Street Institute of Child Health, Guilford Street, London WC1N 1EH, UK; 19 Human Genetics Department, Medical Research Institute, Alexandria University, El-Hadra Alexandria 21561, Egypt; 20 North East Thames Regional Genetics Laboratory, Great Ormond Street Hospital for Children NHS Foundation Trust, Queen Square, London WC1N 3BH, UK; 21 Department of Medical and Molecular Genetics, Division of Genetics and Molecular Medicine, King's College London School of Medicine, Guy's Hospital, London SE1 9RT, UK; 22 Department of Paediatric Respiratory Medicine, National Heart and Lung Institute, Imperial College London, London SW3 6LR, UK; 23 Population, Policy and Practice, University College London (UCL) Great Ormond Street Institute of Child Health, Guilford Street, London WC1N 1EH, UK; 24 Institute of Genetics and Genomics in Geneva, iGE3, CH-1211 Geneva, Switzerland; 25 Host Defence Unit, Respiratory Medicine, Royal Brompton Hospital, London SW3 6NP, UK; 26 Rhinology and Primary Ciliary Dyskinesia Unit, General and University Hospital, Medical School, Valencia University, Valencia E-46014, Spain; 27 UOSD Laboratorio Analisi Venezia, ULSS12 Veneziana, 30121 Venezia, Italy; 28 The Wellcome Trust Sanger Institute, Wellcome Trust Genome Campus, Hinxton CB10 1HH, Cambridge, UK; 29 Department of Pathology, King Abdulaziz Medical City, Riyadh, 14611 Saudi Arabia; 30 Department of Cardiovascular Medicine and Wellcome Trust Centre for Human Genetics, Roosevelt Drive, Oxford OX3 7BN, UK; 31 University of Cambridge Metabolic Research Laboratories and NIHR Cambridge Biomedical Research Centre, Institute of Metabolic Science, Addenbrooke's Hospital, Cambridge CB2 0QQ, UK; 32 Dubowitz Neuromuscular Centre, UCL Institute of Child Health and Great Ormond Street Hospital, London, WC1N 3JH, UK; 33 MRC Human Genetics Unit, MRC Institute of Genetic and Molecular Medicine, Western General Hospital, University of Edinburgh, Edinburgh EH4 2XU, UK; 34 Cardiovascular Genetics, BHF Laboratories, Rayne Building, Institute Cardiovascular Sciences, University College London, London WC1E 6JJ, UK; 35 University College London (UCL) Genetics Institute (UGI) Gower Street, London, WC1E 6BT, UK; 36 Department of Medical Genetics, Cambridge Institute for Medical Research, University of Cambridge, Cambridge CB2 2XY, UK

## Abstract

By moving essential body fluids and molecules, motile cilia and flagella govern respiratory mucociliary clearance, laterality determination and the transport of gametes and cerebrospinal fluid. Primary ciliary dyskinesia (PCD) is an autosomal recessive disorder frequently caused by non-assembly of dynein arm motors into cilia and flagella axonemes. Before their import into cilia and flagella, multi-subunit axonemal dynein arms are thought to be stabilized and pre-assembled in the cytoplasm through a DNAAF2–DNAAF4–HSP90 complex akin to the HSP90 co-chaperone R2TP complex. Here, we demonstrate that large genomic deletions as well as point mutations involving *PIH1D3* are responsible for an X-linked form of PCD causing disruption of early axonemal dynein assembly. We propose that PIH1D3, a protein that emerges as a new player of the cytoplasmic pre-assembly pathway, is part of a complementary conserved R2TP-like HSP90 co-chaperone complex, the loss of which affects assembly of a subset of inner arm dyneins.

## Abstract (web summary)

Primary ciliary dyskinesia (PCD) is a genetically heterogeneous disease resulting in reduced mucus clearance and impaired lung function. Here, the authors show that mutations in PIH1D3 are responsible for an X-linked form of PCD, affecting assembly of a subset of inner arm dyneins.

In mammals, motile cilia are abundant (200–300 per cell) in multiciliated cells lining various specialized epithelia, including the airways, the oviduct and ependyma. The motile cilium is highly similar in structure to the motile flagella of sperm, being a long cellular projection sustained by a complex cytoskeleton structure (axoneme) composed of >200 proteins. Motile cilia axonemes are composed of nine peripheral microtubule doublets surrounding two central microtubules (9+2) except for motile cilia of the embryonic node, which lack the central apparatus (9+0). Several other structures attach along the length of the microtubules at 96-nm intervals to coordinate axonemal beating, including ATP-hydrolysing motor protein complexes called the outer dynein ‘arms' (ODAs) and inner dynein arms (IDAs), as well as radial spokes and nexin–dynein regulatory complexes^1^.

Structural or ciliogenesis defects that affect cilia motility cause primary ciliary dyskinesia (PCD), a generally autosomal recessive disorder affecting 1 per 10,000–15,000 live births^2^,^3^. Typical features are chronic and progressive respiratory symptoms arising from deficient mucociliary clearance of the airways, manifesting in diffuse congestive lung disease and bronchiectasis. Since sperm flagella are structurally similar to cilia, PCD has a high rate of male infertility. Laterality defects arising from dysmotile nodal cilia affect half of patients, causing situs inversus or often more complex heterotaxies and congenital heart defects^4^. PCD is clinically and genetically heterogeneous with mutations in >30 genes causing disease^2^. A common defect is loss of both ODAs and IDAs from the ciliary axonemes, due to mutations in genes encoding axonemal dynein assembly (‘DNAAF') and attachment factors including DNAAF1/LRRC50 (refs 5, 6), DNAAF2/KTU^7^, DNAAF3 (ref. 8), DNAAF4/DYX1C1 (ref. 9), DNAAF5/HEATR2 (refs 10, 11), SPAG1 (ref. 12), C21ORF59 (ref. 13), LRRC6 (refs 14, 15), ZMYND10 (refs 16, 17) and CCDC103 (refs 18, 19). The joint loss of the ODAs and IDAs is one of the most variable ultrastructural defects underlying PCD since affected individuals often have partial defects and truncated dynein arms^7^,^9^,^12^,^13^,^16^,^19^.

The assembly of dynein arms into cilia is a highly evolutionarily conserved mechanism within the cytoplasm. Dynein components synthesized in the cell body are pre-assembled into multi-subunit ‘arm' structures, which are transported by intraflagellar transport (IFT) into the axoneme for microtubule attachment^20^,^21^. The stabilization, folding and pre-assembly of the dynein arm motors is governed by a cytoplasmic chaperone-mediated protein network^20^,^22^,^23^. ODA assembly is thought to involve chaperone-mediated attachment of intermediate chain (IC) dyneins DNAI1 and DNAI2 to heavy chain (HC) dyneins (DNAH5, DNAH11). This IC–HC assembly is facilitated by DNAAF1, DNAAF2 and DNAAF3, with DNAAF3 probably acting at the final stages of chaperone dissociation^8^. DNAAF2 binds at least one IC (DNAI2), heat shock protein (HSP) 70 (ref. 7), as well as DNAAF4 which itself can bind both HSP70 and HSP90 (refs 9, 24). DNAAF2–DNAAF4 were recently described as an R2TP-like co-chaperone complex, similarly recruited to HSP90 for assembly and regulation of multi-subunit complexes^25^,^26^. Other cytoplasmic axonemal dynein assembly factors are also emerging to have likely chaperone functions, for example C21ORF59 (ref. 27).

Here, by studying the molecular basis of PCD in unexplained cases, we have identified a new player in this cytoplasmic pre-assembly pathway, mutations in which cause X-linked disease and a variable loss of cilia dynein arms in both human PCD and the corresponding zebrafish PCD model. From structural, functional and clinical electron tomography data, we propose this might act in a complementary module akin to the DNAAF2–DNAAF4–HSP90 co-chaperone complex.

## Results

### *PIH1D3* mutations cause PCD

**Figure 1:**
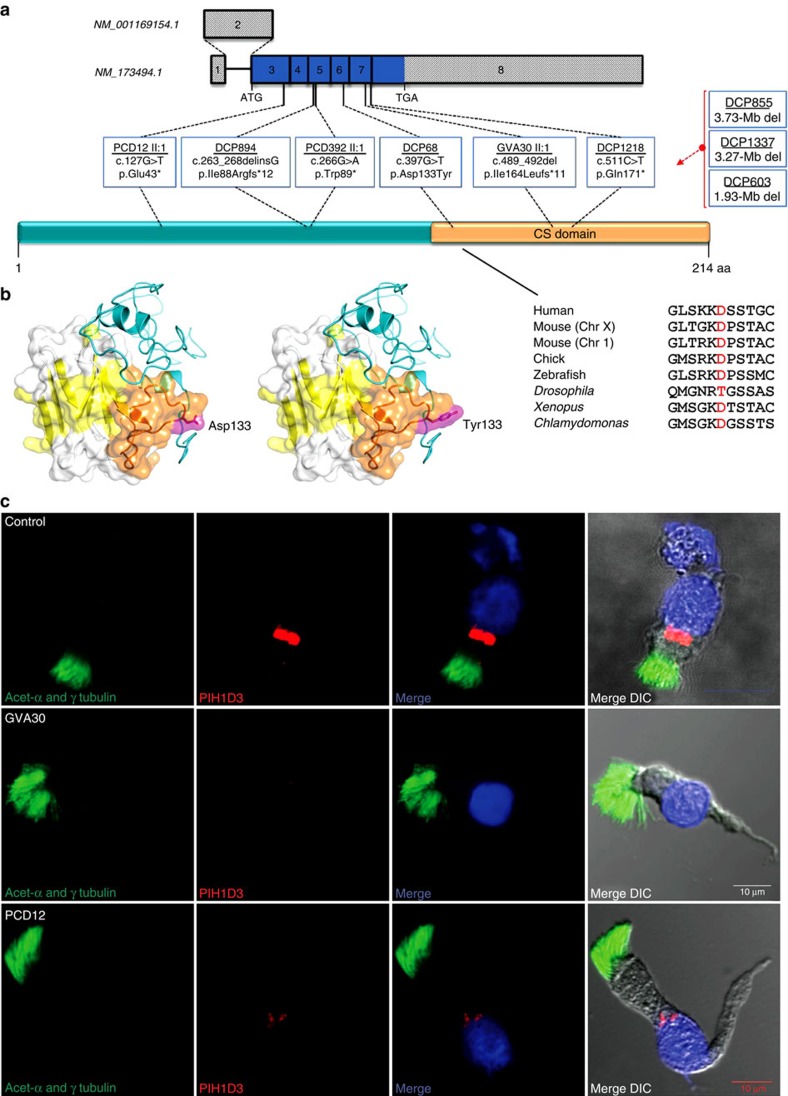
Location of X-linked *PIH1D3* mutations and reduced cytoplasm-localized PIH1D3 protein in airway epithelia of affected individuals. (**a**) *PIH1D3 (CXorf41)* on the X chromosome has two alternate transcripts NM_001169154.1 and NM_173494.1 differing by the presence of an extra untranslated exon (exon 2) in NM_001169154.1. The open reading frame is contained in exon 3–8 (blue boxes). The *PIH1D3* mutations identified are shown within the 214 amino acid protein, in relation to its sole identifiable functional domain, a CS (named after CHORD-containing proteins and SGT1) domain-spanning residue 128–214 (ScanProsite). Mutations are labelled according to the first affected male screened per family. (**b**) Cross-species conservation of the mutated amino acid Asp133 (D133) is shown beneath the protein, in red (right), and PHYRE-based^63^ three-dimensional modelling of the whole of human PIH1D3 shows a surface representation of the highly conserved C-terminal CS domain, within which the Asp133 is predicted to be a surface residue (in magenta) (left). Also highlighted are the beta sheet of the CS domain (yellow), the loops within the CS domain that join the beta strands (white) and the long loop within the CS domain on which Asp133 is located (orange). In dark blue, the disordered N terminus lacks any known structural domains. The effect of the mutated Tyr133 residue is shown (middle). (**c**) Immunofluorescence confocal microscopy analysis of PIH1D3 (red) in dissociated airway (nasal) epithelial cells from controls showed specific localization within the cell body in proximity to the nucleus (DAPI, blue) and no co-localization with ciliary basal body or axonemal markers (γ-tubulin and acetylated-α tubulin, respectively, green) (upper panel). In cells from *PIH1D3*-mutated individuals GVA30 II:1 and PCD12 II:1 (middle and lower panels) there is marked reduction of PIH1D3 protein staining with undetectable levels in GVA30 II:1. Differential interference contrast (DIC) microscopy shows the outline of the cells. Scale bars, 10 μm.

A number of approaches were used by three molecular genetics laboratories (London, Geneva and Paris) to identify novel mutations causing PCD: next-generation sequencing (NGS) of whole exomes of affected individuals from 76 families, a targeted gene panel in affected individuals from 133 families and whole-genome SNP-array analyses in a male patient with intellectual disability and two male siblings from a consanguineous union. The identification of putative mutations in the X-linked gene *PIH1D3* in different patients prompted us to screen this gene by Sanger sequencing in a cohort of 32 independent male patients; this targeted screening identified a high proportion of affected families, with four males from three families carrying *PIH1D3* mutations (9.5% (3/32) of independent cases screened). Overall, these studies led to the identification of *PIH1D3* molecular defects in affected males from nine independent families, which were all family-unique (Fig. 1a; Supplementary Figs 1 and 2): three nonsense mutations (c.127G>T, p.Glu43* in PCD12 II:1; c.266G>A, p.Trp89* in PCD392 II:1; c.511C>T, p.Gln171* in DCP1218), two frameshift mutations (c.263_268delinsG, p.Ile88Argfs*12 in DCP894; c.489_492del; p.Ile164Leufs*11 in GVA30 II:1), one missense change (c.397G>T, p.Asp133Tyr in DCP68), as well as genomic deletions containing the *PIH1D3* gene (1.93-Mb, 3.27-Mb and 3.73-Mb deletions in DCP603/DCP1747, DCP1337 and DCP855, respectively). The complement of genes contained within the latter, larger deletions are listed in Supplementary Table 1. In the NGS screening no other variants of interest were detected, and all the nine identified *PIH1D3* variants were unique, having never been previously reported and being absent from all available databases of normal human variation (ExAC, 1000G, EVS). The nonsense, frameshift and deletion mutations are all predicted as protein disrupting and Fig. 1b shows a high level of conservation of Asp133 which is located within a key surface domain of PIH1D3, as described further below.

The affected families are of European origin except for PCD392 from Sri Lanka and the affected brothers DCP603 and DCP1747 from the sole consanguineous family (DC393) who are from Morocco (Supplementary Fig. 1; Supplementary Table 2). Familial segregation analysis by Sanger sequencing showed inheritance consistent with recessive X-linked disease in all cases, an inheritance pattern never before shown for non-syndromic PCD (Supplementary Fig. 1). Over-expressed mutated GFP-tagged PIH1D3 showed results consistent with the expected effect on the protein (Supplementary Fig. 3). Of the total 13 affected males, 6 had situs inversus totalis which is consistent with the typical PCD randomized left–right body plan. All have classic PCD features including neonatal respiratory distress, recurrent respiratory infections, chronic cough, sinusitis, recurrent pneumonia, otitis media and bronchiectasis (Supplementary Table 2). PCD392 II:1 had developmental delay considered unrelated to PCD and the affected individuals DCP855 and DCP1337 carrying larger chromosomal deletions of >3 Mb affecting multiple genes also had intellectual disability. Sperm motility tested in five affected men carrying *PIH1D3* mutations showed that all had static flagella (asthenozoospermia) (Supplementary Table 2).

### PIH1D3 is a putative axonemal dynein assembly factor

As a first step in investigating the likely function of PIH1D3, we looked at the domain organization of the protein. PIH1D3 is predicted with high confidence not to contain a ‘PIH' domain (‘protein interacting with heat shock protein 90'), but rather to contain a highly conserved ‘CS' fold occupying 40% of the protein (Fig. 1a; Supplementary Fig. 4). PIH and CS are structurally distinct domains, although found in functionally similar proteins. The CS is a bipartite protein–protein interaction domain (Interpro #007052) so called because it is shared by CHORD (cysteine- and histidine-rich domain)-containing proteins and the HSP90 co-chaperone SGT1 (suppressor of the G2 allele of Skp1)^9^,^7^,^28^. CS domains are also found in other HSP90 co-chaperones and protein folding proteins^29^. In contrast, the N-terminal domain of PIH1D3 lacks any identifiable domain and is predicted with high confidence to be disordered, that is, without fixed structure, although it contains several strongly conserved regions that might have functional importance perhaps through binding to partner proteins (Supplementary Fig. 4).

To further investigate the possible role of PIH1D3 in cilia motility, we performed cross-species BLAST analyses, showing that PIH1D3 is a highly conserved protein present only in species that have motile cilia/flagella containing dynein arms and those that require IFT for ciliary assembly (Supplementary Fig. 5). This supports a role for PIH1D3 in IFT-related dynein arm assembly. Phylogenetic evolutionary analysis showed that while humans have a single X-linked copy of *PIH1D3*, mice have two homologous copies located on chromosomes 1 and X (Supplementary Fig. 6). The intronless Chr. 1 mouse gene is most likely an expressed pseudogene recently diverged from and younger than the presumed ancient, intron-containing Chr. X gene. The proteins expressed from the murine Chr. 1 and Chr. X copy are 91% identical, differing mainly in the unstructured N terminus (Supplementary Fig. 4b). Knockout mice lacking the exclusively testis-expressed intronless mouse chromosome 1 pseudogene (that has been termed *Pih1d3*, 4930521A18Rik) have immotile sperm flagella with dynein arm defects and male sterility, but notably do not manifest situs inversus, respiratory cilia dysfunction, hydrocephalus or any ciliary phenotypes typical of PCD^30^. The testis-specific *Pih1d3* is not detectable in the mouse node at day 8, when embryonic symmetry is first broken, and this correlates with the knockouts never showing situs inversus^30^. The knockouts still express the mouse X-linked gene (called *Pih1h3b*, E230019M04Rik), which is expressed more widely than testis, also in the lung, brain and oviduct, but is evidently not able to rescue the sperm flagella defect arising from loss of the Chr. 1 copy^30^. This evolutionary evidence shows a role for PIH1D3 in dynein arm assembly, a role that appears to have recently diverged in mice being governed by different proteins expressed in mouse sperm flagella and cilia, with the cilia-expressed X-linked copy unable to compensate for the Chr. 1 copy in sperm. This contrasts with our data in humans showing that X-linked mutations in the sole human *PIH1D3* gene cause both cilia and sperm defects.

To further investigate the likely function of PIH1D3, we determined where the protein is expressed in cells using a PIH1D3-specific antibody (demonstrated in Supplementary Fig. 3) and high-resolution immunofluorescence confocal microscopy imaging in ciliated respiratory epithelial cells from healthy controls and *PIH1D3* mutation carriers. A specific localization within the cytoplasm was detected with strong staining of PIH1D3 observed in proximity to the nucleus, but no co-localization with ciliary basal body or axonemal markers (γ-tubulin and acetylated-α tubulin, respectively). The cytoplasmic localization of PIH1D3 further supports its putative role in PCD as a cytoplasmic axonemal dynein assembly factor^5^,^6^,^7^,^8^,^9^,^10^,^15^. Notably, in cells from *PIH1D3*-mutated individuals GVA30 II:1 (p.Ile164Leufs*11) and PCD12 II:1 (p.Glu43*) a marked reduction of PIH1D3 protein staining was seen, with very faint staining in PCD12 II:1 and undetectable levels in GVA30 II:1 (Fig. 1c).

### *PIH1D3* mutations confer variable dynein arm defects

**Figure 2:**
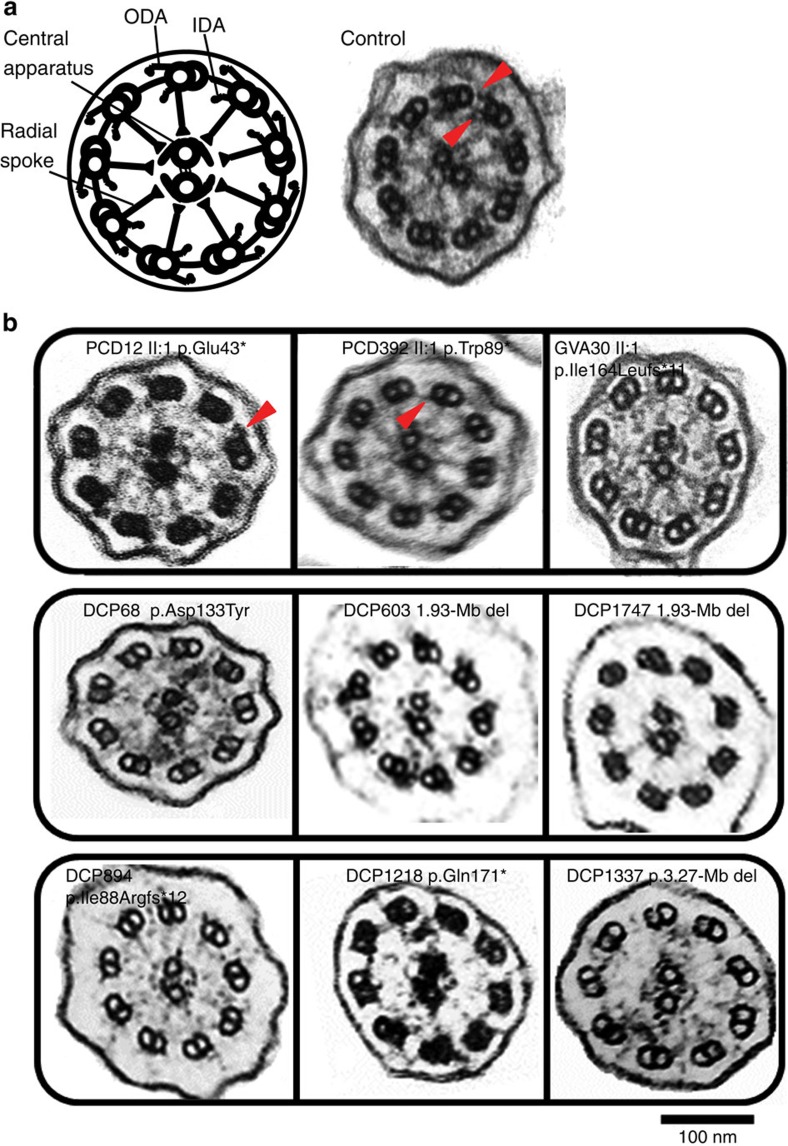
PIH1D3 mutations result in non-assembly and loss of IDAs and ODAs in respiratory cilia axonemes. (**a**) TEM showing the cross-section arrangement of cilia from a healthy control (right) and an image (left) of the main structures of the 9+2 motile axoneme including the outer (ODA) and inner (IDA) dynein arms (red arrowheads). (**b**) TEM of PIH1D3-mutant cilia from nasal respiratory epithelial cells of affected individuals display a reduction and loss of both the ODAs and IDAs. All cases show a combined loss of ODAs and IDAs but this defect can be variable and in particular mutations p.Glu43* (p.E43*, in PCD12 II:1) and p.Trp89* (p.W89*, in PCD392 II:1) are associated with some visible retention of the arms, in particular the outer (PCD12) or inner (PCD392) dynein arms (red arrowheads). In comparison, all other mutations result in the majority of cross-sections showing complete loss of both ODAs and IDAs. These images represent TEM data surveys from a minimum of 300 cross-sections per patient sample.

We next investigated the effect of *PIH1D3* mutations at the ultrastructural level by transmission electron microscopy (TEM) of *PIH1D3*-mutated respiratory cilia, performed within two clinical centres employing multiple replicates analysing >300 cilia cross-sections per replicate. This revealed in all available cases but to variable degree, a loss of both the ODAs and IDAs (Fig. 2; Supplementary Table 2). The spectrum of dynein arm loss arising from PIH1D3 mutation comprised cilia from GVA30 II:1, DCP855, DCP1337, DCP603, DCP1747, DCP894, DCP1218 and DCP68 displaying an almost complete loss of both ODA and IDA in available samples with hardly any normal cross-sections apparent, whilst PCD392 II:1 (p.Trp89*) displayed comparatively less IDA loss still with extensive loss of ODAs (Fig. 2b). However, PCD12 II:1 (p.Glu43*) appeared to be an unusual outlier, displaying much less ODA loss and primarily an IDA loss phenotype (Fig. 2b).

**Figure 3:**
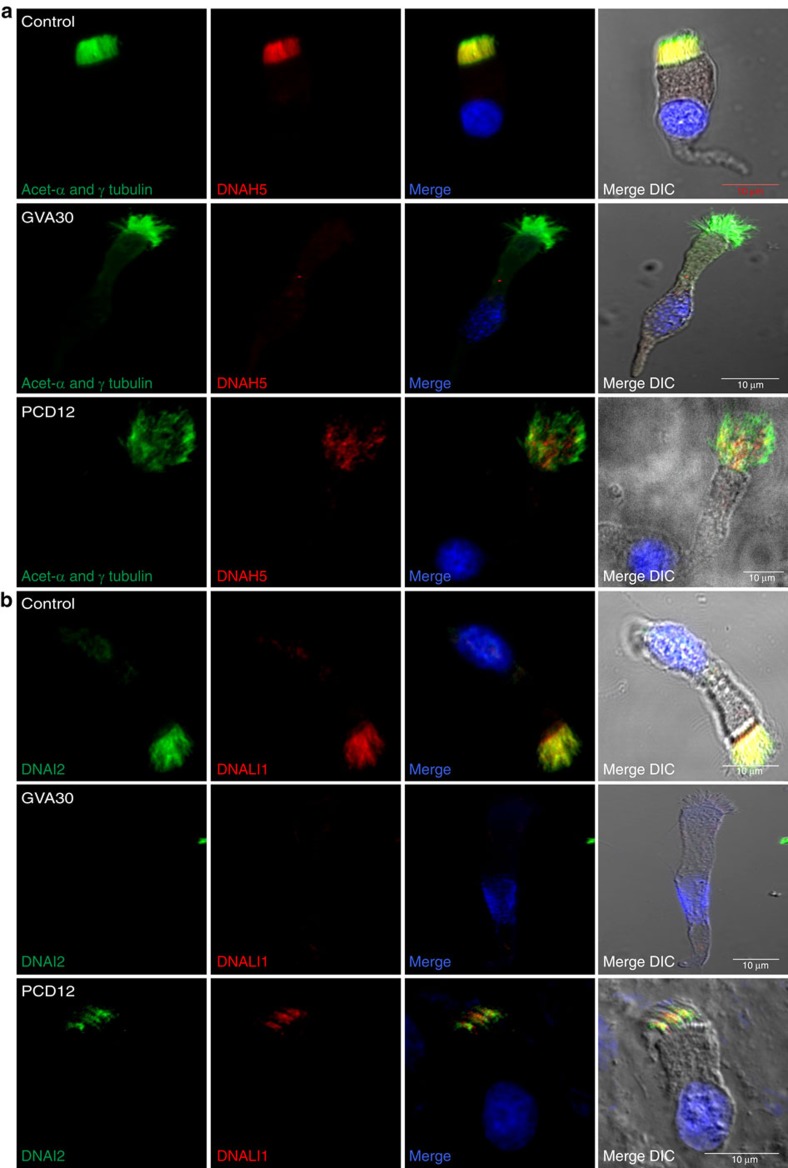
PIH1D3 mutations cause defective dynein arm assembly in respiratory cilia. (**a**) Respiratory epithelial cells from control and PCD-affected individuals GVA30 II:1 and PCD12 II:1 carrying *PIH1D3* mutations were double-labelled with antibodies directed against acetylated α-tubulin and γ-tubulin (green) to mark the ciliary axonemes and basal bodies, and DNAH5 as a marker of the ODA (red). Both α-tubulin and DNAH5 colocalize (yellow) along the full length of the cilia axonemes in cells from the unaffected control (upper panel). In contrast, in cells from affected individuals GVA30 II:1 (p.Ile164Leufs*11) and PCD12 II:1 (p.Glu43*) (lower panels), DNAH5 is highly reduced in the ciliary axonemes compared with α-tubulin, being undetectable in GVA30 II:1 and markedly reduced in PCD12 II:1. (**b**) Respiratory epithelial cells from control and PCD-affected individuals GVA30 II:1 and PCD12 II:1 carrying *PIH1D3* mutations were also double-labelled with antibodies directed against another ODA marker DNAI2 (green) and the IDA marker DNALI1 (red). Both proteins colocalize (yellow) along the full length of the cilia axonemes in cells from the unaffected control (upper panel). In contrast, in the *PIH1D3* mutants, both DNAI2 and DNALI1 are reduced in the ciliary axonemes, being undetectable in GVA30 II:1 and markedly reduced in PCD12 II:1. DIC shows the cell outline. Scale bars, 10 μm.

We proceeded to investigate the effect of *PIH1D3* mutations on assembly of the ODAs and IDAs at the molecular level by immunofluorescence staining of *PIH1D3*-mutated respiratory cilia using antibodies directed against two established markers of human dynein arms, the dynein HC ODA marker DNAH5 and dynein light IC IDA marker DNALI1. DNAH5 staining was reduced in *PIH1D3*-mutated individuals, being undetectable in the axonemes of GVA30 II:1 (p.Ile164Leufs*11), corroborating with the TEM evidence of disrupted ODA assembly (Fig. 3a). In PCD12 II:1 (p.Glu43*) staining was greatly reduced as well, but some retention of DNAH5-stained ODAs was apparent, which is consistent with the TEM data shown in Fig. 2. Another ODA marker, the intermediate dynein chain DNAI2, and DNALI1 were analysed and found to display similar reduced levels in the cilia axonemes of GVA30 II:1 (p.Ile164Leufs*11) and PCD12 II:1 (p.Glu43*), again with more apparent retention of both in PCD12 II:1 (Fig. 3b). This supports the TEM findings that PCD12 II:1 retains more of the ODAs than GVA30 II:1, and also some IDAs.

The variable retention of ODAs and IDAs in *PIH1D3*-mutated cilia mirrors the variable effects of different mutations in some other known dynein assembly factor PCD genes^7^,^9^,^12^,^13^,^16^. In the case of PCD12 II:1 (p.Glu43*), in spite of the *PIH1D3* nonsense mutation there appears to be less severe loss of ODAs than that of the other patients, suggesting a less severe mutation. The involvement of modifying genetic factors cannot be excluded in this individual; alternatively, the resulting *PIH1D3* transcripts may escape, at least in part, degradation via the nonsense-mediated mRNA decay (NMD) pathway and the protein may retain some degree of normal function. In keeping with this hypothesis, the Human Splicing Finder V3.0 tool (http://www.umd.be/HSF3/index.html) predicts that the c.127G>T mutation identified in PCD12 II:1 causes the loss of a potential exonic splice enhancer (ESE) at the 3′ end of exon 3 (Supplementary Fig. 7). This mutation is expected to alter splicing, especially since the MaxEntScan application^31^ predicts the strength of the physiological intron 3 donor splice site to be very low (that is, score of 1.07, as compared with 7.97 to 11 for the other *PIH1D3* donor splice sites). On the basis of this hypothesis, we performed RT-PCR on total RNA from a limited amount of nasal biopsy material obtained from PCD12 II:1, which confirmed the absence of exon 3 from *PIH1D3* transcripts compared with those amplified from nasal biopsy of a healthy control (Supplementary Fig. 8). This indicates that the *PIH1D3* c.127G>T mutation causes an exon skipping event in PCD12 II:1 that removes exon 3 containing the normal ATG initiation codon as well as the p.Glu43* nonsense mutation. This could allow the use of an in-frame alternative ATG initiation codon, downstream of the usual start codon located at the beginning of exon 3. The corresponding transcripts would therefore escape NMD and a shorter in-frame PIH1D3 protein could be expressed. Such an alternative ATG indeed exists in exon 4 (codon 61) and it contains a good Kozak consensus sequence as predicted by ATGpr^32^ (score of 0.15 versus 0.16 for the usual ATG initiation codon).

We investigated *PIH1D3*-mutated cilia motility using high-speed video microscopy imaging. All affected individuals had immotile cilia consistent with dynein arm loss: as a representative example of these patients, only entirely static cilia could be seen in PCD392 II:1 (Supplementary Video 1), while in controls a healthy coordinated beating pattern was observed (Supplementary Videos 2 and 3). However in PCD12 II:1 (p.Glu43*) the cilia retained some movement, displaying within the same sample a mixed beating pattern consisting of patches of static cilia alongside patches of cilia beating with normal frequency but a jittery dysmotile and disorganized pattern (Supplementary Videos 4 and 5). Since ODAs govern cilia beat frequency and propagate asymmetric waveforms, whilst IDAs regulate cilia waveform and propagation^33^,^34^, we speculate that the static cilia patches in PCD12 II:1 (p.Glu43*) correspond to those more lacking in ODAs, whilst the cilia with retained but disorganized beating could be those more lacking in IDAs.

### PIH1D3 has a conserved ciliary role in other vertebrates

**Figure 4:**
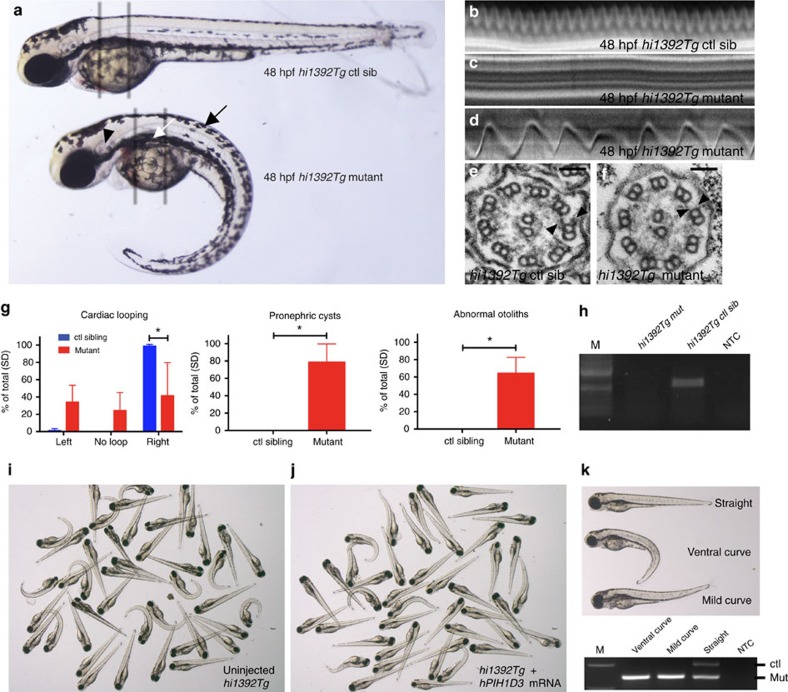
Conserved function for PIH1D3 in vertebrate cilia motility and dynein arm assembly replicating the human disease. (**a**) Comparison of a representative *hi1392Tg* control sibling (ctl sib, top), and a *hi1392Tg* mutant (bottom) harbouring a transgenic insertion in the *pih1d3* (previously known as *twister*) gene, at 48 h post fertilization (48 hpf). Mutants display an abnormally curved body axis (black arrow), pronephric cysts (white arrow), abnormal otolith formation (black arrowhead). (**b**–**f**) Cilia defects are evident in *hi1392Tg* mutants at 48 hpf, with the grey bars in **a** highlighting areas used for pronephric cilia imaging shown in **b**–**f**. Cilia motility defects are evident in representative kymographs recorded in *hi1392Tg* mutant siblings (**c**,**d**) compared with *hi1392Tg* control siblings (**b**), created from 0.5-s, 1,000 fps videos of single pronephric cilia bundles beating in 48 hpf (representative of 16 recordings). Mutants had mainly static cilia (**c**) with an occasional cilia showing retained reduced beating (**d**). Also see Supplementary Videos 6,7,8,9. (**e**,**f**) TEM of 48 hpf pronephric cilia cross-sections from the same area reveal a reduction and loss of outer and IDAs (arrowheads) in *hi1392Tg* mutants (**f**) compared with control siblings where the arms are visible (**e**). Scale bar, 50 nm. (**g**) Quantification of common phenotypes of *hi1392Tg* mutant embryos (red columns) compared with control sibling embryos (blue columns). *hi1392Tg* mutant embryos display 64–84% levels of abnormal (that is, absent or reverse) cardiac looping, pronephric cysts and otolith malformations, while this was never, or rarely, seen in control siblings. Otolith malformation was recorded if there were more or less than two observed. Columns represent data from three separate experiments assessing phenotype in 48 hpf embryos (*n*=40–71 per experiment). Error bars show standard deviation with *t*-test, *P*-value *<0.05. (**h**) The insertion *hi1392Tg* creates a null allele: RT-PCR of a pool of 15 embryos indicates a complete lack of *pih1d3* transcript expression in *hi1392Tg* mutants compared with a pool of 15 control siblings. (**i**–**k**) Examples of clutches of 72 hpf embryos from *hi1392Tg/+* crosses injected at one-cell stage with 10 pg of human *PIH1D3* mRNA show a reduction in mutant phenotypes (**j**) compared with non-injected *hi1392Tg* control siblings (**i**), with representative embryos with a straight, ventral curve, or mild curve phenotype shown in **k**, upper panel. PCR genotyping in all three of these groups shows the lower *hi1392Tg* insertion allele band present, but the upper control allele band is present only in the straight-tailed siblings (**k**, lower panel). M, DNA size marker; NTC, non-template control.

We further investigated PIH1D3 function using zebrafish *pih1d3*^*hi1392Tg/hi1392Tg*^ mutants generated by transgenic insertion. Previous work identified a link in these mutants between loss of PIH1D3 and cystogenesis within the glomerular-tubular region of pronephric kidneys, but did not directly test for ciliary defects^35^. We found that zebrafish possess a single orthologous gene *pih1d3* 46% identical to human *PIH1D3* (Supplementary Fig. 4b). RT-PCR confirmed that *pih1d3* mRNA is maternally deposited in zebrafish, and is further expressed during the first 2 days when several ciliated organs become present (Supplementary Fig. 9). *hi1392Tg* mutants consistently developed cysts in pronephric glomeruli and tubuli (Fig. 4a,g). This phenotype was previously linked to a lack of pronephric fluid flow caused by defective pronephric cilia motility^36^, and is exhibited in *hi1392Tg* mutants with visible pronephric dilation and dispersal of the normally closely packed pronephric cilia (Supplementary Fig. 10)

We found that cilia are virtually immotile in *hi1392Tg* mutants, both in pronephric tubuli and olfactory bulbs (Fig. 4b–d; Supplementary Videos 6,7,8,9). TEM also revealed absent ODAs and IDAs in mutant cilia, thus phenocopying human PIH1D3-mutated patients (Fig. 4e,f). The *hi1392Tg* mutants additionally displayed consistent ventral tail curvature that is typical for PCD zebrafish models, and two other phenotypes considered to result from cilia dysmotility: abnormal cardiac looping presumed to arise from defective Kupffer's vesicle cilia motility^37^ and abnormal otolith formation caused by otic vesicle cilia dysmotility^38^ (Fig. 4g). RT-PCR showed a complete lack of expression of the *hi1392Tg* allele (Fig. 4h), and injecting zebrafish *pih1d3* mRNA completely or largely rescues the phenotypes of ventral body axis curvature, pronephric cysts and abnormal cardiac looping (Supplementary Fig. 11).

To test whether PIH1D3 is functionally conserved from zebrafish to human, we injected human *PIH1D3* mRNA into embryos from crosses of heterozygous carriers of the *hi1392Tg* allele. Overexpression of the human protein in zebrafish appears to be detrimental. By titrating the dosage down to 10 pg, the ventral curvature phenotype is partially rescued in some of the mutant embryos (Fig. 4i–k).

### PIH1D3 acts early in dynein assembly with DNAI2 interaction

**Figure 5:**
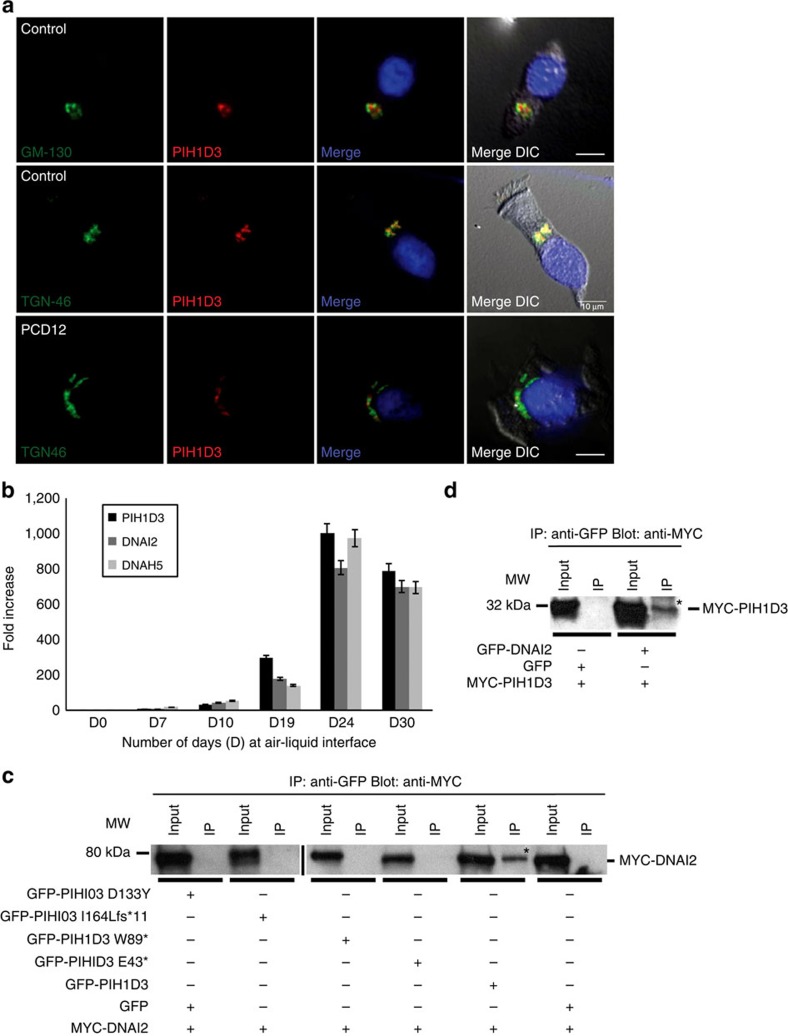
Golgi-localised PIH1D3 is upregulated during ciliogenesis and forms a complex with DNAI2 destroyed by disease-causing mutations. (**a**) Immunofluorescence confocal microscopy analysis in dissociated airway (nasal) epithelial cells from controls shows no co-localization of PIH1D3 (red) with the *cis*-Golgi marker GM-130 (top panel, green). However, co-localization is seen using the *trans*-Golgi marker protein TGN-46 (middle panel, green), implying PIH1D3 is present within the late rather than early/medial Golgi. The affected individual PCD12 II:1 showed no gross disruption of *trans*-Golgi staining as a result of PIH1D3 mutation using co-localization studies of TGN-46 (green) and PIH1D3 (red) (bottom panel). Nuclei in blue (DAPI). DIC shows cell outline. Scale bars, 10 μm. (**b**) Quantitative RT-PCR analysis of *PIH1D3* in normal human bronchial epithelial cells grown on an ALI to follow their progression from nonciliated basal cells to multiciliated epithelia over 30 days (D) in culture. Day (D) 0 is the first day the cells are exposed to the ALI, and typically cilia first become visible in these cultures from day 17. The qPCR analysis compared expression of *PIH1D3* to the *DNAH5* and *DNAI2* genes, mutations in which are known to cause PCD. A similar increase in mRNA expression of all three genes was seen over time in ALI culture, from around day 10 with a peak at day 24. Around day 19, the apparent higher increase in PIH1D3 levels was not significant. The fold increase in each gene's expression was measured in triplicate, normalized at each time point to its expression at day 0. Error bars indicate s.e.m. (**c**) Co-IP assays were performed on human embryonic kidney (HEK293) cells co-transfected with an expression vector encoding Myc-tagged DNAI2 and either an empty GFP vector or GFP-tagged PIH1D3, corresponding either to normal full-length PIH1D3 protein or mutant PIH1D3 protein carrying mutations identified in affected individuals. Anti-GFP antibody IP was performed using a standard protocol followed by western blotting using anti-Myc antibody, showing that Myc-DNAI2 co-precipitates only with wild-type GFP-PIH1D3 and not with GFP-PIH1D3 harbouring any of the four identified disease mutations (p.Asp133Tyr/p.D133Y, p.Ile164Leufs*11/p.I164Lfs*11, p.Trp89*/p.W89* and p.Glu43*/p.E43*). (**d**) A reciprocal co-transfection of Myc-tagged wild-type PIH1D3 into HEK293 cells with either empty GFP vector or GFP-tagged DNAI2 followed by anti-GFP IP and anti-Myc western blotting confirmed the co-IP between Myc-PIH1D3 and GFP-DNAI2. MW, molecular weight marker. kDa, kilodaltons. Asterisks in **c**,**d** indicate the immunoprecipitated proteins (labelled on the right). Uncropped blots can be found in Supplementary Fig. 14.

We sought to further explore PIH1D3 ciliary function using the human antibody that detects a specific protein product around the Golgi region of the cytoplasm, near the nucleus (Fig. 1c). High-resolution immunofluorescence analysis revealed that PIH1D3 localizes to the *trans*-Golgi network in ciliated epithelia cells, with a high degree of specific co-localization between PIH1D3 and the late Golgi marker TGN-46 but much less colocalisation with the *cis*-Golgi marker GM-130 (Fig. 5a). Co-localization with TGN-46 was also confirmed after overexpression of PIH1D3 in HEK293 cells (Supplementary Fig. 12). PCD12 II:1 (p.Glu43*)-ciliated airway epithelial cells did not show TGN disruption or loss of TGN-46 co-localization because of their depleted PIH1D3 protein (Fig. 5a). Thus, any retained protein expressed from this allele localizes normally. Expression of *PIH1D3* compared with two other PCD genes (*DNAH5*, *DNAI2*) during ciliogenesis in cultured-ciliated human airway epithelial cells showed a similar profile, increasing before emergence of the motile cilia around day 10, with peak expression around day 24 falling to a lower plateau from day 24. Co-expression of *PIH1D3* with other PCD genes supports our proposal that it has a shared function (Fig. 5b).

To further investigate the role of PIH1D3 in dynein assembly, we examined its interactions with DNAI2, an ODA intermediate dynein chain co-assembled with another IC [DNAI1] during the first steps of cytoplasmic ODA pre-assembly^7^,^30^,^39^. Co-expression of GFP-tagged human PIH1D3 and Myc-tagged human DNAI2 in HEK293 cells followed by co-immunoprecipitation (IP) showed an interaction between the two proteins (Fig. 5c), which was also confirmed with swapped tags (Fig. 5d), supporting a role for PIH1D3 in axonemal dynein assembly. Since modelling predicts that the Asp133Tyr missense mutation identified in patient DCP68, which replaces a small charged residue (Asp) with a polar aromatic residue (Tyr), is within the CS in a high confidence conserved predicted surface of PIH1D3 that mediates protein–protein interactions (Fig. 1b; Supplementary Fig. 4a,b), we tested whether introduction of this missense mutation could alter the ability of PIH1D3 to interact with DNAI2. We found that the PIH1D3–DNAI2 interaction was destroyed by introduction of the missense mutation (Fig. 5c). Correspondingly, p.Asp133Tyr is predicted as 1.0 ‘probably damaging' using Polyphen-2 (ref. 40) and 0.05 ‘deleterious' according to SIFT^41^, consistent with a high level conservation in multiple ciliate species across Asp133 (Fig. 1b; Supplementary Fig. 4b). Similar results were obtained with three other deleterious (nonsense, frameshift) human *PIH1D3* mutations identified in this study (Fig. 5c), indicating the functionally destructive nature of all four DNA variants that were modelled.

### Mutations in *PIH1D3* can result in partial IDA assembly

**Figure 6:**
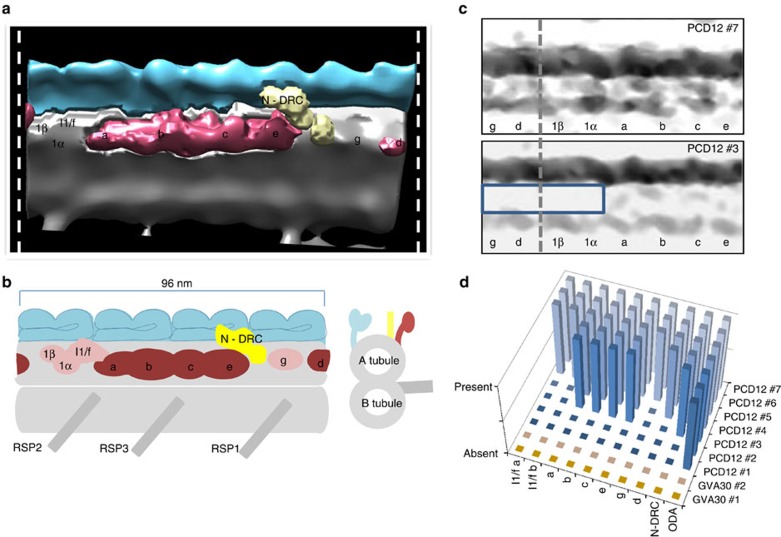
***PIH1D3*** **mutations cause a failure of specific dynein components to assemble into the** IDA. (**a**) Electron tomography based three-dimensional map of the axonemal 96 nm repeat (indicated by dashed white lines) generated from an averaged tomogram (tomogram PCD12 #4 which is also shown in **d**), from individual PCD12 II:1 harbouring the *PIH1D3* mutation p.Glu43*. The data reconstruction displays the IDA coloured using Chimera software, showing the absence of the α and β subunits of dynein I1 (also known as f), in addition to dynein g, as explained in **b**. (**b**) A diagram of the 96 nm repeat of the human axoneme shown in both longitudinal (left) and transverse (right) section, based on imaging of normal tomograms (see Supplementary Fig. 13) and current published literature. ODAs are depicted in blue, IDAs in red with the inner arm dynein components absent from individual PCD12 II:1 shown in a lighter pink shade. The nexin link/dynein regulatory complex (N-DRC) is shown in yellow, with radial spoke (RSP) complexes 1, 2 and 3 and the anchoring microtubule doublets in grey. (**c**) Electron density analysis along the 96 nm repeat (boxed), showing in longitudinal section the presence of all inner and ODA components from PCD12 tomogram #7 and the absence of the I1/f α, I1/f β, d and g subunits (boxed) from PCD12 tomogram #3, taken from the tomograms represented in **d**. Dashed line shown for orientation compared with **a**. (**d**) Representation of the presence or absence of IDA axonemal components in different tomograms taken from patients PCD12 II:1 (tomograms #1–#7) and GVA30 II:1 (tomograms #1, #2). IDAs were not present in the axonemes of cilia from GVA30 II:1, whilst there was more variability in PCD12 II:1 and IDA partial loss always involved non-assembly of dyneins I1/f, I1/f and g.

Having placed human PIH1D3 into the earliest steps of the DNAI2-ODA cytoplasmic assembly process alongside DNAAF2, a finding also supported by previous work in mice^30^, we next sought higher resolution understanding about the dynein arm defects in *PIH1D3*-mutated human cilia. We used electron tomography averaging to create a three-dimensional map of the axonemal 96 nm repeat containing the entire IDA, from available tissue of PCD12 II:1 (p.Glu43*) and GVA30 II:1 (p.Ile164Leufs*11). Fig. 6a,b shows a representative image of PCD12 II:1 (p.Glu43*), compared with a healthy control (Supplementary Fig. 13). There were generally few IDAs available for imaging, but we were able to detect them in some sections. In imaging of GVA30 II:1, tomograms only captured full absence of the whole IDA along with absent N-DRCs (nexin–dynein regulatory complex) and ODAs (Fig. 6d). However, imaging of PCD12 II:1 revealed three different patterns of IDA assembly in the tomograms, with ODAs being present in all imaging. The entire IDA plus N-DRC were either entirely present (Fig. 6d, PCD12 #5–#7), entirely absent (Fig. 6d, PCD12 #1 and #2) or else a partial IDA assembly was seen. Partial IDA assembly found in 2 out of 7 tomograms (Fig. 6d, PCD12 #3 and #4) showed a consistent pattern of loss of the α and β subunits of IDA dynein I1 (also known as f) and IDA dynein g. Dynein d loss was undetermined, being either present or absent (Fig. 6a,d). Electron density mapping further confirmed the presence of all IDA and ODA components except I1/f α, I1/f β and the d and g subunits in tomogram #3 (Fig. 6c). This partial IDA assembly is consistent with *Chlamydomonas* data suggesting different cytoplasmic axonemal dynein assembly factors might specifically assembly distinct sets of IDA dynein subunits^22^.

## Discussion

Here we report different approaches taken across three centres to identify the cause of disease in a large cohort of PCD patients. This identified protein-altering mutations in *PIH1D3*, a previously uncharacterized X-linked gene in humans. X-linked transmission of PCD was previously suggested^42^ and *RPGR* mutations can cause syndromic X-linked PCD with retinitis pigmentosa^43^, but *PIH1D3* mutations are the first reported molecular cause of X-linked non-syndromic PCD. *PIH1D3* mutations may be a prominent cause of X-linked PCD, since a small targeted screen in this study for mutations in affected males with PCD due to lack of dynein arms so far unexplained at the molecular level detected *PIH1D3* mutations in 9.5% of cases.

The affected males carrying *PIH1D3* mutations displayed deficient mucociliary clearance, situs inversus and asthenozoospermia, indicating that in humans the single X-linked *PIH1D3* participates in axonemal dynein assembly in both the respiratory system and testis, as well as in laterality determination. In contrast mice have two *PIH1D3* homologues, a testis-restricted mouse Chr. 1 *Pih1d3* being essential for dynein assembly in the sperm flagellum, while mouse Chr. X *Pih1h3b* is expressed in testis and also in ciliated tissues (lung, brain, oviduct) but cannot compensate to rescue sperm motility. In the Human Protein Atlas^44^ human PIH1D3 is expressed in ciliated tissues, being highest in lung, fallopian tube and testis, supporting our evidence that PIH1D3 function in humans, unlike in mice, is not split between two isoforms.

Several other lines of evidence in this study reveal the possible biological role for PIH1D3 in cilia and flagella assembly. *PIH1D3* expression is upregulated in parallel with other PCD genes during human respiratory epithelial ciliogenesis. PIH1D3 is exclusively cytoplasmic in multiciliated respiratory epithelial cells, a localization disrupted in *PIH1D3*-mutated cilia. PIH1D3 is highly conserved, as confirmed by recapitulation of structure and motility defects in *PIH1D3*-mutated human cilia and in *hi1392Tg* zebrafish *pih1d3*-null mutants, and the phenotypic rescue of zebrafish mutants by human *PIH1D3*. Affected boys and the zebrafish model both show that *PIH1D3* mutations result in loss of ODAs and IDAs. Ultrastructural studies detected some variation in the degree of ODA versus IDA loss in different patients carrying *PIH1D3* mutations, which might be explained by their different genetic backgrounds or by different severity of specific mutations. Its cytoplasm localization and link to loss of dynein arms indicate that PIH1D3 is part of the same pathway as axonemal dynein assembly factors including DNAAF2–DNAAF4 that are likely co-chaperones of HSP90 (refs 25, 26). Mutations in DNAAF2 and DNAAF4 also cause PCD with joint ODA and IDA loss. We have shown that like DNAAF2, PIH1D3 interacts *in vitro* with DNAI2, an intermediate dynein representing one of the first components of the human ODA assembly process.

Clinical tomography imaging of the entire IDA shows both complete or partial loss of IDA dyneins in PIH1D3-mutant cilia. Little is known about human IDA assembly but similarly to *Chlamydomonas*, the human IDA contains seven major species of dyneins labelled a–g^45^,^46^. We find in PIH1D3-mutant cilia where there is a partially assembled IDA, that the IDA dynein subunits f and g specifically fail to assemble. Selective assembly of different IDA dynein subunits by different assembly factors was previously hypothesized in *Chlamydomonas*^22^. Our data indicating that specific assembly of dynein f and g (but not a or c) is under the control of PIH1D3, possibly in a complementary manner to IDA dynein assembly by DNAAF2, supports a differentiation in the pathways of human axonemal dynein assembly, whereby different assembly factors assemble different subunits and PIH1D3 works alongside DNAAF2 to assemble complementary sets of IDA dyneins.

**Figure 7:**
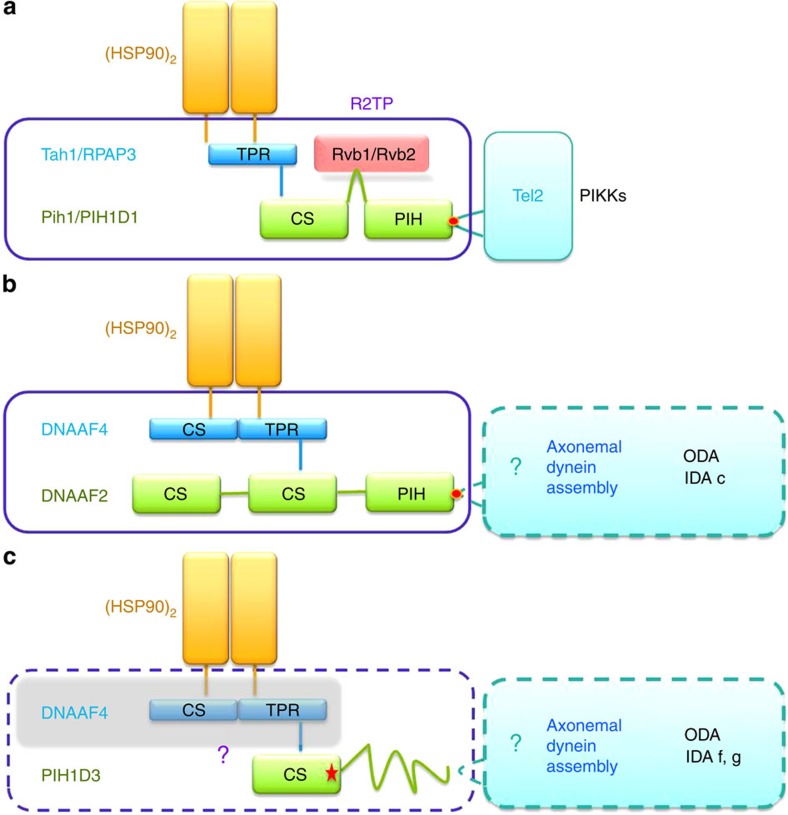
Proposed model of PIH1D3 function in HSP90-mediated axonemal dynein assembly. PIH1D3 may participate in an R2TP-like co-chaperone complex composed of TPR (tetratricopeptide repeat), CS (CHORD-containing proteins and SGT1) and PIH1 (protein interacting with heat shock protein 90)-containing proteins to chaperone HSP90 for pre-assembly of axonemal dyneins in the cytoplasm, before their IFT-associated import into motile cilia and flagella. The proposed model compares PIH1D3 to two other cilia dysmotility-associated CS/PIH proteins mutated in PCD, DNAAF2 and DNAAF4 which are known to interact. (**a**) The yeast R2TP complex forms a multi-protein co-chaperone (purple box, human orthologs also shown) that recruits HSP90 dimer for activation of phosphoinositol-kinase like kinases (PIKKs) through a novel phosphorylation motif (red spot) in Tel2 that associates with the PIH domain of Pih1. (**b**) A structurally homologous co-chaperone DNAAF2/DNAAF4 module is proposed that may recruit HSP90 and HSP70 to axonemal dynein assembly. The phospho-motif binding site is conserved in the DNAAF2 PIH domain (red spot), although the phosphorylated partner is unknown. (**c**) CS domain similarity between PIH1D3 and DNAAF2 shows PIH1D3 may participate in a distinct co-chaperone module also required for axonemal dynein assembly. PIH1D3 does not contain a PIH domain, therefore, a different motif may be used to link to other proteins within the dynein assembly pathway—the unstructured N terminus of PIH1D3, shown as a ribbon, is predicted to become structured on binding of a protein partner. The PCD-associated mutation p.Asp133Tyr (red star) within the CS domain disrupts interaction between PIH1D3 and DNAI2, an early component of axonemal dynein assembly. It is plausible that like DNAAF2, PIH1D3 could serve its function by an interaction with DNAAF4 (grey box). Biochemical analysis in *Chlamydomonas* and tomography data presented here suggest DNAAF2 and PIH1D3 are both involved in ODA assembly, and also in assembly of specific IDA dyneins. Boxes with solid lines represent known complexes and boxes with dashed lines possible complexes. ODA, outer arm dyneins; IDA, inner arm dyneins.

Our combined structural and functional data have highlighted a ciliary role for PIH1D3 in assembly of both the IDAs and ODAs. Recent analysis suggesting two known assembly factors DNAAF2 and DNAAF4 might form an R2TP-like complex for dynein assembly^26^ helps further define the possible function of PIH1D3 within this pathway (Fig. 7). First described in yeast, the R2TP co-chaperone regulates essential HSP90-related functions to direct the assembly of many multi-subunit complexes, including the phosphatidylinositol-3-kinase-like kinases (PIKKs) for signal transduction^23^. HSP90 is a molecular chaperone that folds and activates several hundred client proteins involved in many cellular processes, and its specificity of action is determined by numerous co-chaperones that regulate its ATPase activity and recruit the proteins it is required to fold^47^,^48^. In human R2TP, PIH1D1 acts as an adaptor linking HSP90 and RPAP3 (Tah1 in yeast) to RUVBL1 and RUVBL2 (yeast Rvb1/Rvb2) (Fig. 7a)^23^. The PIH domain of yeast Pih1 (human PIH1D1) was recently resolved as a phosphopeptide-binding domain that associates with a novel phosphorylation motif (DpSDDxF) in Tel2 to regulate PIKKs (Fig. 7a)^26^. Recruitment of R2TP to HSP90 is mediated through the C terminus CS domain of Pih1/PIH1D1 by TPR protein Tah1/RPAP3 that binds a conserved motif (MEEVD) of HSP90.

CS/TPR-containing DNAAF4 and CS/PIH-containing DNAAF2 are similar to the TPR domain protein RPAP3 and the CS/PIH protein PIH1D1, and evidence that the two interact with each other and with HSP90 and HSP70 (refs 9, 24) suggests they could form an R2TP-like co-chaperone complex participating in axonemal dynein assembly^25^,^26^ (Fig. 7b). The phospho-motif binding site identified in Pih1/PIH1D1 (ref. 26) is conserved in the DNAAF2 PIH domain, indicating a similar function in mediating phosphorylation-dependent protein–protein interactions, though the phosphorylated partner is unknown (Fig. 7b, red dot). The interaction of DNAAF2 with DNAI2 could indicate its link into the dynein assembly pathway (Fig. 7b). In the case of PIH1D3 (Fig. 7c), sequence and structural homology between its CS domain and that in DNAAF2 suggests that it could also interact with HSP90 though a TPR-containing intermediary (possibly but not necessarily DNAAF4), thereby acting as a co-chaperone recruiting HSP90 chaperone function into axonemal dynein assembly. The p.Asp133Tyr missense change that disrupts PIH1D3 interaction with DNAI2 confirms the functional importance of this residue within the PIH1D3 CS domain that might link directly to dynein assembly or indirectly via another assembly factor (Fig. 7c, red star).

By inference to the mouse testis-specific PIH1D3 homologue^30^, it is possible that human PIH1D3 is not associated with DNAAF1, DNAAF2 and DNAAF3, and that it functions separately during the assembly of dynein arm complexes. We find PIH1D3 is essential for assembly of the ODAs, and for IDA assembly it appears more strongly associated with assembly of only specific IDA subunits, the IDA dyneins f and g (Fig. 7c). *Chlamydomonas* biochemistry has previously suggested that DNAAF2 specifically assembles ODAs and IDA dynein subunit c (Fig. 7b)^7^. Our data appear to confirm some complementarity amongst human assembly factors as well, since isolated loss of dynein c was never seen in the PIH1D3-mutant cilia of PCD12 II:1 (Fig. 6). The remaining human IDA dyneins could be assembled specifically by other assembly factors and no human ortholog has yet reliably been identified for MOT48, which is a third PIH-domain cytoplasmic dynein assembly factor of *Chlamydomonas* that appears to assemble its other IDA dyneins, although MOT48 overlaps somewhat with DNAAF2 since it is thought to specifically assemble IDA dyneins b, c, d and e^22^,^30^.

In conclusion, we propose that PIH1D3 is a cytoplasmic protein involved in early axonemal dynein arm assembly as a likely HSP90 co-chaperone participating to assemble ODAs and certain IDA dynein subunits. PIH1D3 mutations affect its interaction with protein partner(s) in dynein assembly and give rise to a classic form of PCD disease with dynein arm loss. PIH1D3, therefore, joins the family of human cytoplasmic PIH/CS dynein assembly factors, probably acting in a complex in a similar manner to the putative DNAAF2–DNAAF4 co-chaperone. We propose that it could be re-named DNAAF6 to prevent confusion about its structural domains. Better understanding of dynein assembly factors and their chaperone-mediated interactions in PCD may provide an important target for therapy in future. Our findings of X-linked PCD have clinical importance for improved counselling of individuals affected by PCD and their families, in what has up until now been described as a largely autosomal recessive condition. *PIH1D3* should be prioritized for mutational screening in PCD cases lacking dynein arms and in cases of PCD with a suggestive X-linked inheritance pattern.

## Methods

### Patients and sample collection

Individuals included in the study had a clinical diagnosis of PCD confirmed by standard clinical diagnostic criteria documenting typical symptoms of neonatal respiratory distress and chronic respiratory disease features including rhinosinusitis, re-occurring airway infections and productive cough, otitis media and bronchiectasis. Clinical test results included nasal nitric oxide measurements, medical imaging, in addition to light, electron and immunofluorescence microscopy to detect ciliary motility and analyse ciliary structure. For studies of affected individuals and their families, signed and informed consent was obtained from all participants before history recording, blood drawing and nasal biopsy, using protocols approved by the ethics review boards of the University Medical School in Geneva (Switzerland), the Institute of Child Health/Great Ormond Street Hospital, London (UK) (#08/H0713/82) and the Comité de Protection des Personnes CPP Ile-de-France III (France) (approvals no. CPP07729 and CPP02748).

### Next-generation sequence analysis

DNA was extracted from whole blood of affected individuals and family members by standard methods, with automated platforms such as Qiagen Autopure LS and NucliSENS used for the Geneva cohort. In the UK, the PCD12 sample was included in a cohort of 43 affected individuals subject to whole-exome sequencing as part of the UK10K project^49^. PCD392 was one of 48 affected individuals screened by targeted sequence capture. This used the SureSelectXT system (Agilent) and sequence capture libraries designed using the Agilent SureDesign wizard, to capture the full transcript of 640 target genes plus 25 basepairs (bp) at each intron–exon boundaries. A total of 25 ng of genomic DNA was fragmented with QXT enzyme and tagged with adaptors for initial amplification. Libraries were captured and sequencing performed using standard protocols for the Illumina HiSeq platform (paired ends, 2 × 150 bp). Barcoding allowed multiplexing of eight cases per flow cell. Sequence data was analysed using an in-house pipeline^50^ with sequence reads in FASTQ format aligned to the reference human genome (hg19) using BWA (0.6.1-r104) and default settings^51^. Variant calling was performed across the entire region of interest using VarScan2 (V.2.3.7) set at minimum 30 × coverage, minimum five alternate reads, minimum phred-like base-quality of 20 (ref. 52). Variant calls in VCF format were then annotated using Ensembl Variant Effect Predictor (V.73) and the output parsed using an in-house script, which converts the annotated VCF file into Excel format for subsequent variant filtering and prioritization^53^. In Geneva, genomic DNA of 59 affected individuals (belonging to 47 families) was fragmented by sonication to 200 bp fragments then liquid phase captured against whole human exome probes to select all coding sequences (SureselectXT Target Enrichment System Version 1.2, Agilent). Sequencing was performed on an Illumina HiSeq 2000 instrument (paired ends, 2 × 95 bp). Each exome library was indexed, separated into two equal halves and sequenced in two different lanes, up to five half-libraries were sequenced in each HiSeq lane. The raw results were analysed using a local bioinformatics pipeline, which as previously described^16^ combines published algorithms in a sequential manner (BWA for map reads, SAMtool for detection of variants, Pindel for the detection of indels, ANNOVAR for annotation of sequence changes). The entire coding sequence corresponding to RefSeq (http://www.ncbi.nlm.nih.gov/refseq/) coding genes was used as the reference for the calculation of coverage and reads on target.

In both centres, for subsequent filtering of variants, only exonic and splicing variants (±10 bp of the intron–exon boundary) were retained for further analysis. These were filtered to exclude synonymous variants, variants with a minimum allele frequency ≥0.01 in dbSNP version 137, 1000Genomes (http://1000genomes.org/), local databases and variants found within segmental duplications of the genome. Minor allele frequency was then checked on Exome Variant Server (http://evs.gs.washington.edu/EVS/) and ExAC (http://exac.broadinstitute.org/) databases, as well as in VariantMaster^54^ in Geneva, to further filter for variants common among the affected individuals. Final filtered variants were evaluated individually based on the predicted pathogenicity scores provided by a number of softwares including SIFT, PolyPhen-2 and Mutation Taster, their presence in HGMD (http://www.hgmd.cf.ac.uk/ac/index.php) and the literature, focusing on functional data. Evolutionary conservation scores were provided by PhyloP and GERP++.

### Whole-genome SNP-array analysis

Patients were genotyped for copy number variations with the HumanOmniExpress 24 BeadChip from Illumina and the data were analysed with the Genome Studio and CNV partition 3.1.6 softwares (Illumina).

### Sanger sequencing

Sanger sequencing of custom-designed *PIH1D3* amplicons (NM_001169154) confirmed potentially disease-causing variants in probands and allowed segregation analysis in their families. Primers were designed using Primer 3 (http://primer3.ut.ee/) and are listed in Supplementary Table 3 with PCR conditions available on request. Sequences were analysed and compared with controls using Sequencher software (Gene Codes Corporation, Ann Arbor, MI, USA) in UK patients, Mutation Surveyor (SoftGenetics, LLC) and Staden (http://staden.sourceforge.net/) in the Geneva patients and Seqscape software (Applied Biosystems, Foster City, USA) in the French patients.

### Immunofluorescence analysis

All antibodies used are listed in Supplementary Tables 4 and 5. Respiratory epithelial cells obtained by nasal brush biopsy and suspended in cell culture media were air dried onto glass slides and stored at −80 °C until use. Immunostaining on the slides was performed as described^55^. The cells were incubated with primary antibodies overnight at room temperature at the dilutions listed in Supplementary Table 4, then with secondary antibodies diluted at 1:1,000 dilution for an hour. Cells were finally washed five times with PBS, mounted in ProLong Gold Antifade Mountant with DAPI (ThermoFisher Scientific) and confocal images were taken using a Zeiss LSM 710 (Zeiss, Cambridge, UK). Immunofluoresence analysis in Supplementary Fig. 3 verified a Sigma PIH1D3 antibody by specific detection of PIH1D3 at the expected molecular weight.

### cDNA cloning and site-directed mutagenesis

Fully sequenced human PIH1D3 (clone 5173807; Genbank NM_001169154) and DNAI2 (clone 5752153; Genbank NM_023036) cDNA clones were purchased from Mammalian Gene Collection (GE Healthcare) for Gateway Cloning. PCR products were amplified using PWO high-fidelity Taq Polymerse (Roche), subcloned into Gateway entry vector (ThermoFisher) and recombined with pcDNA-DEST53 (ThermoFisher) and pCMV-Myc (Clontech) Gateway Vector via LR Clonase II encoding GFP and Myc N-terminal epitope tags, respectively.

Primers for mutagenesis were designed according to the QuikChange Primer Design Tool (Agilent Technologies). Tagged plasmids were amplified using KAPA HiFi DNA Polymerase (KAPA Biosystems) according to the manufacturer's directions. Amplified products were gel purified and Dpn1 digested for 2 h before transforming into competent XL1-Blue cells (Stratagene). All cDNA and mutant clones were verified by Sanger sequencing. cDNA cloning and site-directed mutagenesis primers are listed in Supplementary Table 3.

### Cell culture and transfection

Human embryonic kidney cells (HEK293T, American Type Culture Collection) were grown to confluence in DMEM (Life Technologies) supplemented with 10% fetal bovine serum and 1% penicillin–streptomycin antibiotic (Invitrogen). Cultures were plated at a density of 6 × 10^5^ cells per well in a 6-well plate. Cells were transfected with plasmids encoding GFP-tagged cDNA constructs, or both Myc- and GFP- tagged plasmids were co-expressed for co-IP assays. DNA (2 μg) was added to each well with 8 μl of Lipofectamine 2000 reagent (Thermo Scientific) in OptiMEM-1 (Invitrogen) and 4 μg DNA (each at 2 μg) for co-expression studies. Within 48 h, cells were collected in 1 × PBS and lysed in 300 ul of 20 mM Tris–Cl (pH 7.4), 50 mM NaCl and 1% NP40 supplemented with protease inhibitor cocktail (Sigma). Lysates were centrifuged at 16,000*g* for 15 min at 4 °C, aliquoted and stored at −20 °C until use.

### Co-IP and immunoblotting

IP assays were performed using Dynabeads Protein G beads (Novex). Briefly, 20 μl of Dynabeads were washed three times in IP buffer (50 mM Tris 7.4, 150 mM NaCl, 1 mM EDTA, 0.5% sodium deoxycholate and 1% NP40). Approximately 2 mg of co-expressed lysate with protease inhibitor cocktail (Sigma) was added to pre-washed beads to pre-clear on a rotator for 2 h at 4 °C. Subsequently, 50 μl of beads were briefly washed three times in IP buffer and then incubated with 1 μg of mouse IgG anti-GFP (clones 7.1 and 13.1, Roche) antibody at room temperature for 1.5 h. Antibody-bead complexes were gently washed three times in IP buffer before incubating with pre-cleared lysate overnight at 4 °C. Bead complexes were gently washed five times in IP buffer and resuspended in 2 × Laemmli sample buffer (BioRad), 1 μl of 2-mercaptoethanol (Sigma) and heated for 10 min at 95 °C to dissociate the complex from the beads. Beads were separated on a magnet and lysates were then by electrophoresed on 10% SDS–PAGE gels, transferred to nitrocellulose membranes and blocked in 5% skimmed milk, 3% BSA dissolved in PBS-Tween for 2 h at room temperature before immunoblotting overnight at 4 °C with mouse anti-Myc (9E10, ThermoFisher). For immunoblotting, 20 μg of lysate was resuspended in 2 × Laemmli sample buffer (BioRad), 1 μl of 2-mercaptoethanol (Sigma) and heated for 5 min at 95 °C and the protocol continued similarly to the IP assay. Membranes were washed four times (10 min each) then incubated with anti-mouse HRP or anti-sheep HRP secondary antibody for 45 min at room temperature. Membranes were then washed five times and developed by Pierce ECL Western Blotting Substrate (ThermoFisher Scientific). Images were developed on X-ray films (GE Healthcare).

The following antibodies were used: mouse monoclonal anti-GFP (clones 7.1 and 13.1, Roche), mouse monoclonal anti-Myc (1:1,000, clone 9E10, Merck Millipore), rabbit polyclonal anti-PIH1D3 (1:400, HPA051099, Sigma), mouse anti-GAPDH (1:1,500, clone 6C5, Merck Millipore), donkey anti-rabbit HRP antibody (1:5,000, NA934, GE Healthcare) and sheep anti-mouse HRP antibody (1:5,000, NXA931, GE Healthcare).

### Air–liquid interface (ALI) cultures

Normal human bronchial epithelial (NHBE) cells were obtained from Lonza. Cultures were transformed at passage 2 with *Bmi1* and maintained on collagen-coated plates with Bronchial Epithelial Growth Media (Lonza) for 7 days to grow to confluence. Cells were seeded at a density of 5 × 10^5^ onto 24-well collagen-coated transwell inserts (Costar, Corning, USA). When confluent after 48 hr cell the cultures were exposed to an air-liquid interface (ALI) as previously described^56^ by removing medium from their surface (apical chamber) and replacing the medium in the basal chamber with ALI medium (1:1 ratio of DMEM 4.5 g l^−1^ D-glucose:Bronchial Epithelial Growth Media containing 100 nM all-trans retinoic acid (Sigma-Aldrich, UK)). Cells were ALI cultured for the next 30 days.

### Quantitative RT-PCR analysis

Total RNA was isolated from ALI culture pellets using Trizol Reagent (Invitrogen) and used to produce complementary DNA with Omniscript (Invitrogen). RT-qPCR primers were designed using Primer-BLAST (http://www.ncbi.nlm.nih.gov/tools/primer-blast/) to ensure specificity and primer sets were optimized across a range of cDNA concentrations by generating standard and melt curves per gene. All real-time qPCR reactions were carried out with a CFX96 Touch Real-Time PCR Detection instrument (Biorad) with samples set up in triplicates in 20 μl reactions containing 2 × iQ SYBR Green Supermix (Biorad), 3 picomolar of 100 μM primer mix (forward and reverse primers) and 500 ng of cDNA. GAPDH was used as an endogenous control for normalization between target gene expression levels. Data acquisition of the fluorescent signal was performed at the end of the run. Double delta CT calculations were measured as logarithm and then converted to fold change of 100, after untreated control levels were subtracted from target levels. Primers used in RT-qPCR experiments are listed in Supplementary Table 3.

### Zebrafish analysis

All zebrafish (*Danio rerio*) experiments were approved by the Institutional Animal Care and Use Committee (IACUC) of Yale University. Husbandry was carried out according to standard protocols^57^. A human rescue expression vector was created by cloning human *PIH1D3* (PCR primers 5′-CACCAGGATCCATGGAATCTGAAAATATGGAT-3′ and 5′-AGGCTCGAGTCAGAAGAAATTAGCAATATC-3′) into the pCS2+ vector between BamHI and XhoI restriction endonuclease sites. For zebrafish *pih1d3*, total RNA was isolated from wild-type TuAB embryos, followed by cDNA synthesis with High-Capacity cDNA Reverse Transcription Kit (Applied Biosystems). PCR products using *pih1d3*-specific primers 5′-(ggatcccatcgattcg)ATGGAGGGTCTCGCGTCC-3′ and 5′-(actatagttctagaggc)TCAGGTCAGATTAATGCAGTCCA-3′ were cloned using the Gibson assembly kit (NEB) into an EcoRI- and XhoI-digested pCS2+ vector. Plasmids were linearized with NotI, followed by 5′cap mRNA synthesis using the SP6 mMESSAGE mMACHINE kit (Ambion). Purified mRNA was injected at 1 nl into 1-cell stage embryos. The embryos were raised at +28.5 °C until analysis. For genotyping, embryos were lysed with 50 mM NaOH, and analysed by duplex PCR using primers 5′-TTACTACTACAATCCGCGTCCATG-3′, 5′-CAAGGTTTGTCAGGCGATAATAGAT-3′ and 5′-GCTAGCTTGCCAAACCTACAGGT-3′. For mRNA expression, mRNA was purified using Trizol (Invitrogen), followed by cDNA synthesis using the High-Capacity cDNA Reverse Transcription Kit (Applied Biosystems) and PCR with primers against full-length *pih1d3*, 5′-ATGGAGGGTCTCGCGTCC-3′ and 5′-TCAGGTCAGATTAATGCAGTCCATTG-3′. For histology, 50 hpf embryos were anesthetized and fixed with Bouin's fixative overnight at room temperature. Fixed embryos were washed with PBT (PBS and 0.1% (v/v) Tween 20), dehydrated and rehydrated with 100% methanol, washed with PBT and embedded in JB-4 plastic resin (Polysciences). The moulds were cut into 4 μm slices and stained with hematoxylin and eosin. For TEM of zebrafish, embryos were anesthetized and fixed in 3% glutaraldehyde and 1.5% paraformaldehyde in a 0.1 M phosphate buffer (pH 7.4) and subsequently stored in 0.1 M phosphate buffer supplemented with 0.01% sodium azide for transportation. Before TEM, embryos were washed overnight and post-fixed in 1% osmium tetroxide. After dehydration, the samples were embedded in epoxy resin. Thin sections stained with Reynold's lead citrate were imaged with a Jeol 1010 electron microscope. For high-speed microscopy and analysis in zebrafish, embryos were anesthetized with 0.2 ml ml^−1^ Tricaine (Sigma-Aldrich) and mounted on microscope slides in embryo medium. DIC images were acquired through a × 40 water immersion objective on a Zeiss Observer z1 fitted with a Hamamatsu Orca Flash 4.0 V2 high-speed camera. Kymographs were created from 0.5-second 1,000 fps videos using ImageJ software.

### Electron tomography

Nasal brushings were chemically fixed and embedded as previously described^58^. In brief; biopsies were obtained from the inferior turbinate of the nose using a cytology brush. For electron microscopy, samples were fixed in 2.5% glutaraldehyde, washed with cacodylate buffer, post-fixed in osmium tetroxide and rinsed with distilled water, with centrifuging taking place between each step. Epithelial strips were bound with 2% agar and then dehydrated and infiltrated with araldite resin and embedded in araldite. Ciliated areas were selected in survey sections before cutting dark gold-blue ultra thin sections. Ultra thin sections (∼150 nm) were stained with methanolic uranyl acetate and lead citrate and 10 nm gold fidicuals were added to aid correlation of the captured images for tomogram generation.

Images of cilia in longitudinal section were acquired at × 20,000 magnification using a Jeol 1400+ transmission electron microscope with a mid-mount digital camera (AMT16X). Automated Recorder software package v2.7 (Jeol Technology Systems) was used to acquire dual axis tilt sequences between 70° and −70° in 1° increments. Tomograms were then generated using IMOD software created by the University of Colorado^59^,^60^. Particle Estimation for Electron Tomography (PEET) software was used to average and align particles along manually selected microtubules^61^. The outcome of the PEET process is a density map, representing an average 96 nm repeat of the selected tubule in which regular, repeated features are emphasized. The density maps were opened within the UCSF Chimera graphics package^62^. A data threshold was set for each individual map based on achieving a balance between structural detail and background noise. The Segger tool was used to split the volume map into distinct segments which were then isolated and coloured to aid visualization.

### Data availability

All the relevant data are available from the authors upon request.

## Additional information

**How to cite this article:** Olcese, C. *et al*. X-linked primary ciliary dyskinesia due to mutations in the cytoplasmic axonemal dynein assembly factor PIH1D3. *Nat. Commun.* **8,** 14279 doi: 10.1038/ncomms14279 (2017).

**Publisher's note:** Springer Nature remains neutral with regard to jurisdictional claims in published maps and institutional affiliations.

## Supplementary Material

Supplementary Information Supplementary Figures, Supplementary Tables, and Supplementary References.

Supplementary Video 1 PCD392 HSVM side view.

Supplementary Video 2 Control HSVM side view.

Supplementary Video 3 Control HVSM top view.

Supplementary Video 4 PCD12 HSVM side view.

Supplementary Video 5 PCD12 HSVM top view.

Supplementary Video 6 hi1392Tg mutant pronephros.

Supplementary Video 7 hi1392Tg ctl sib pronephros.

Supplementary Video 8 hi1392Tg mutant olfactory placode.

Supplementary Video 9 hi1392Tg ctl sib olfactory placode.
