# Supplementary material for: X-linked primary ciliary dyskinesia due to mutations in the cytoplasmic axonemal dynein assembly factor PIH1D3
Source: Nat Commun. 2017 Feb 8;8:14279. doi: 10.1038/ncomms14279 (PMC5309803; doi:10.1038/ncomms14279)
Supplement: Supplementary Information — Supplementary Figures, Supplementary Tables, and Supplementary References. [file ncomms14279-s1.pdf]

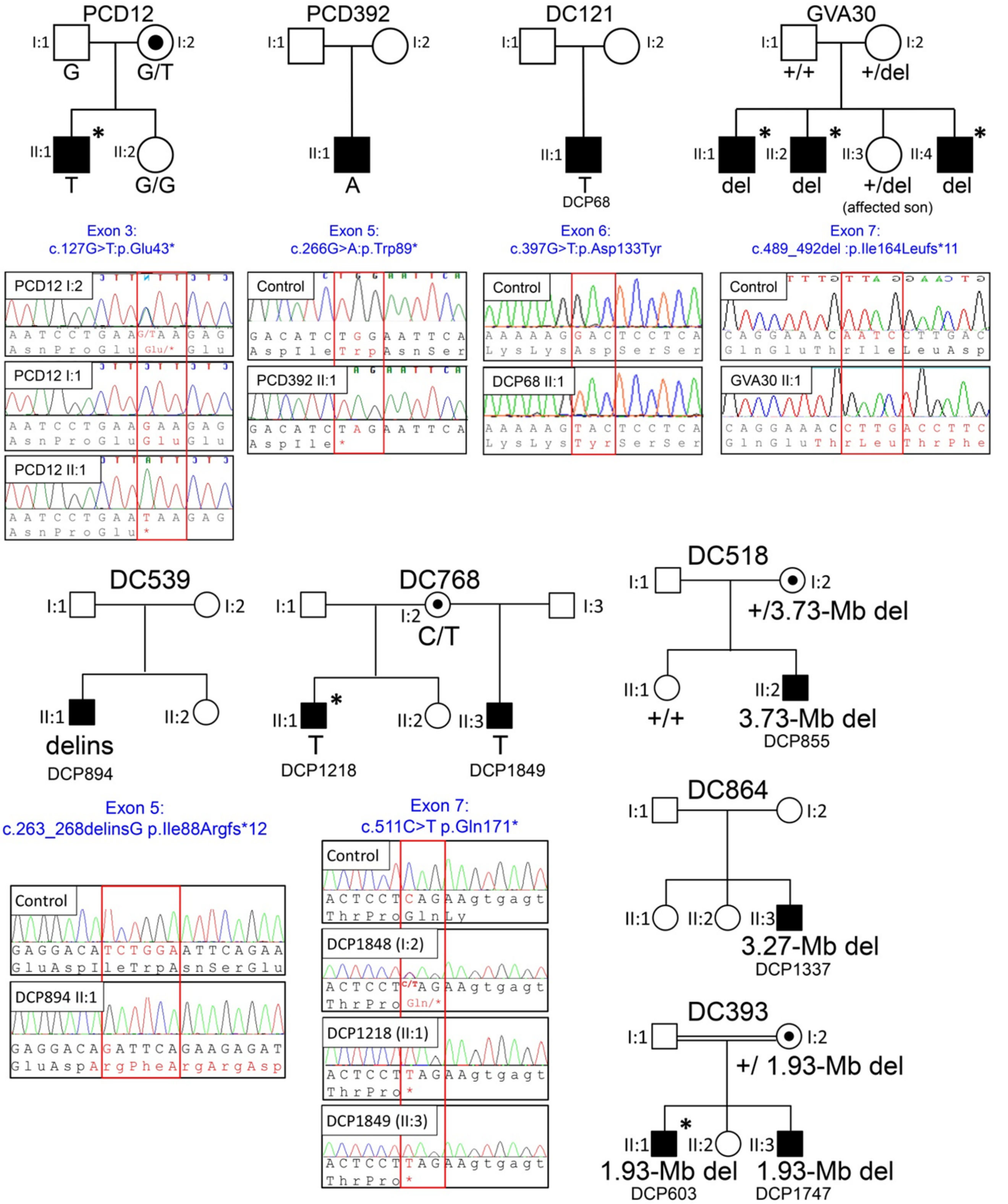

Supplementary Figure 1. Familial segregation analysis of *PIH1D3* mutations

Pedigrees of affected families are shown indicating the genotypes of affected boys (black squares), confirmed carrier females (filled circle), and wildtypes (white boxes and circles). Asterisk indicates situs inversus. In GVA30, carrier sibling II:3 has an affected son (ungenotyped). Probands arrowed. Sanger sequence of mutations are displayed with the mutation in red box and mutant amino acids in red lettering; note that in the GVA30 example, the reverse primer sequence has been used, but the forward sequence content and corresponding amino acids are shown at the bottom.

a

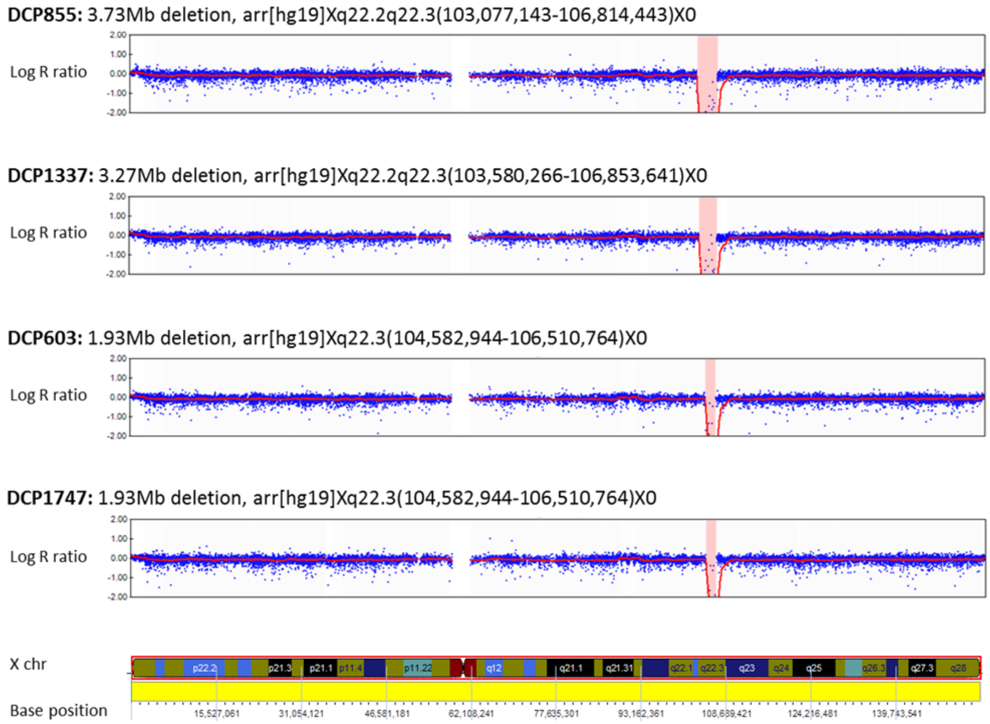

b

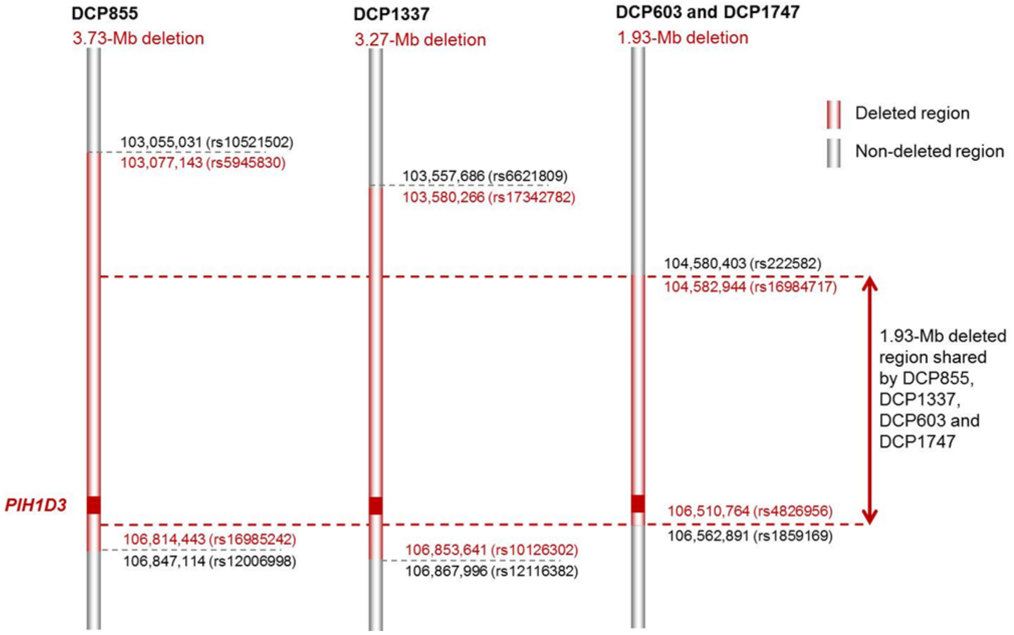

Supplementary Figure 2. Schematic representation of the interstitial deletions of the long arm of the X chromosome carried by affected individuals DCP855, DCP1337, DCP603 and DCP1747

(a) Patients were genotyped with the HumanOmniExpress 24 BeadChip from Illumina and the data were analyzed with the Genome Studio and CNV partition 3.1.6 softwares (Illumina). Each blue dot represents one individual single-nucleotide polymorphism (SNP). Each panel represents the log R ratio (red line), which is the log ratio of observed probe density to expected probe density. The log R ratio drop under 0 (pink area) indicates the loss of material.

(b) SNPs located at the internal and external boundaries of the deletions (Grch37) are respectively in red and black. Red boxes show location of *PIH1D3* at the distal boundary of the 1.93Mb deleted region shared by the four probands.

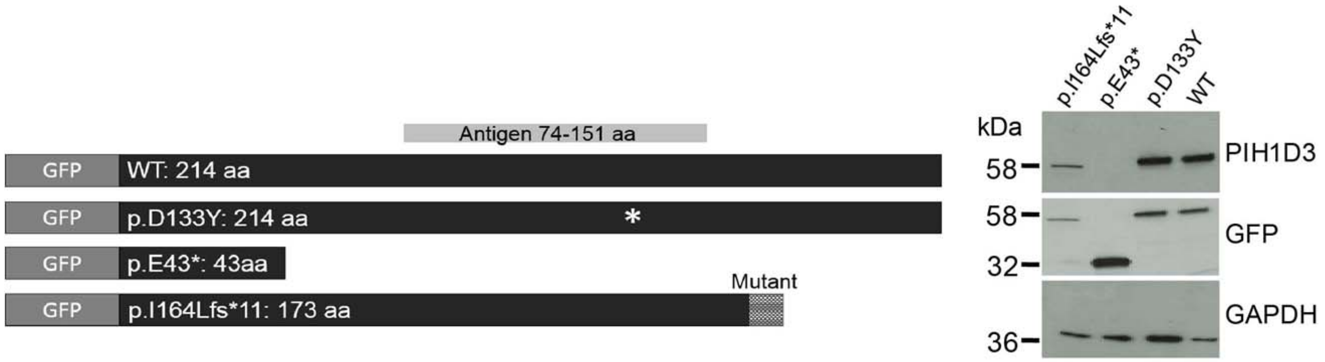

Supplementary Figure 3. Expression of mutant PIH1D3 proteins
In vitro over-expression in HEK293 cells of GFP-tagged human PIH1D3 harbouring different disease-associated mutations. The molecular weight (kDa) of the expressed protein was assessed by western blotting using anti-PIH1D3 (right, top panel), anti-GFP (right, middle panel) or the loading control anti-GAPDH (right, bottom panel) antisera. PIH1D3 carrying the missense change D133Y did not migrate differently, but PIH1D3 carrying p.Glu43* and p.Ile164Leufs*11 were truncated as expected (p.Glu43* protein was only detected by GFP immunostaining since it lacks the domain containing the anti-PIH1D3 antigen, shown left). Uncropped data for the blots is shown in Supplementary Figure 14.

a

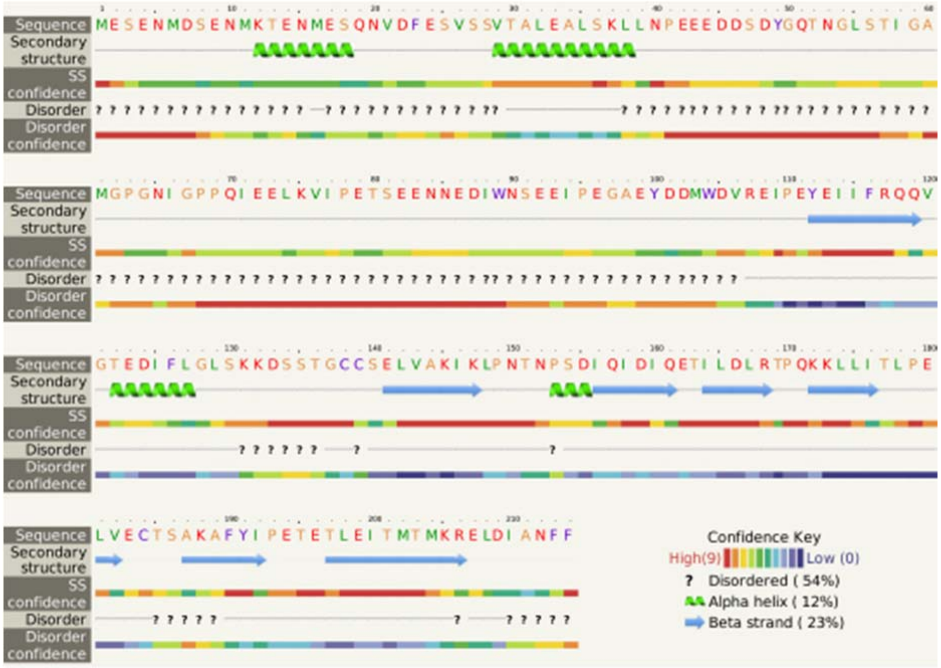

b

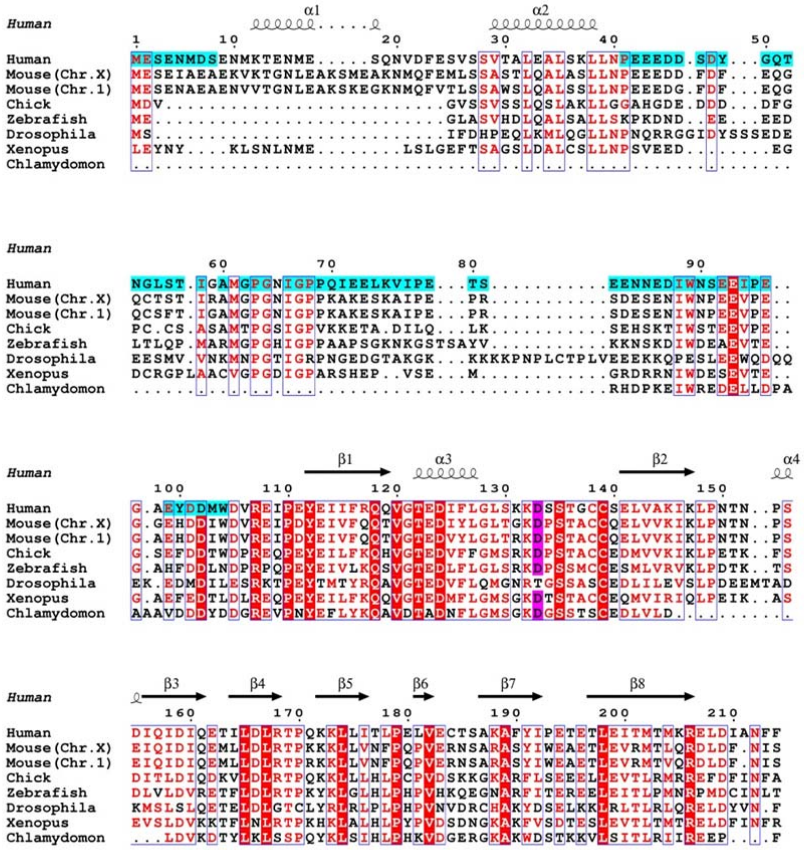

Supplementary Figure 4. PIH1D3 protein modelling and cross-species conservation

(a) Primary structure of PIH1D3 annotated with Phyre-generated secondary structure and disorder, coloured to indicate strength of confidence of domain predictions. (b) Sequence alignment of representative homologues of PIH1D3 generated using T-Coffee (http://tcoffee.crg.cat/), illustrated with ESPript 3.0.3. The Phyre-predicted secondary structure of human PIH1D3 is shown above the alignment. Residues predicted with high confidence to be disordered are coloured cyan. D133 is highlighted in magenta.

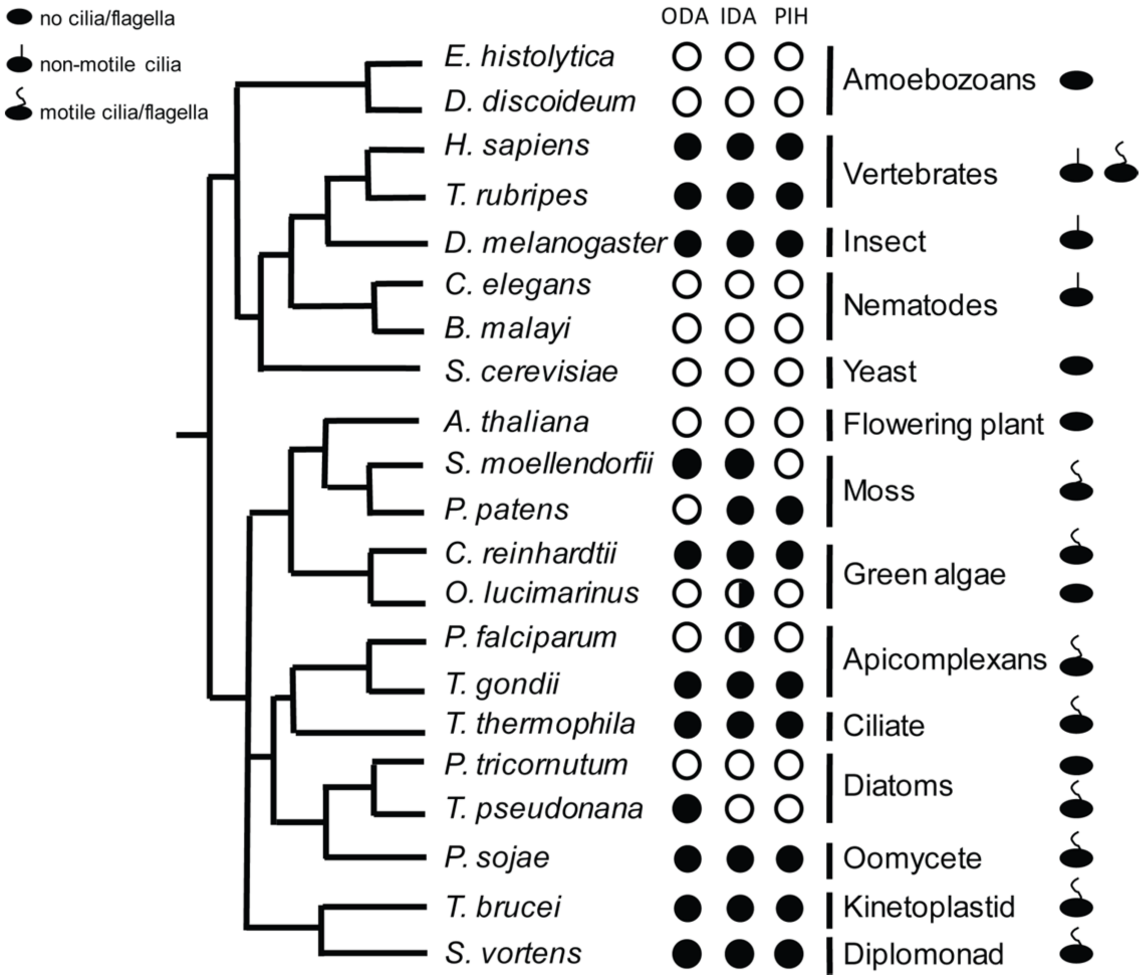

Supplementary Figure 5. Eukaryote conservation of PIH1D3 orthologs across cilate species
Scoring presence (black circle) or absence (white circle) of a PIH1D3 orthologue in species without cilia/flagella, with nonmotile cilia or with motile cilia/flagella. PIH1D3 is present in species with ODA and IDA, supporting a role in intraflagellar transport (IFT)-related dynein arm assembly. Akin to studies on another putative dynein assembly factor, LRRC56, PIH1D3 is absent from the moss *Selaginella moellendorfii* which shows secondary loss of ODAs (its male gametes have flagella only), and also the diatom *Thalassiosira pseudonana* that retains ODAs but does not require IFT for ciliary assembly.[2]

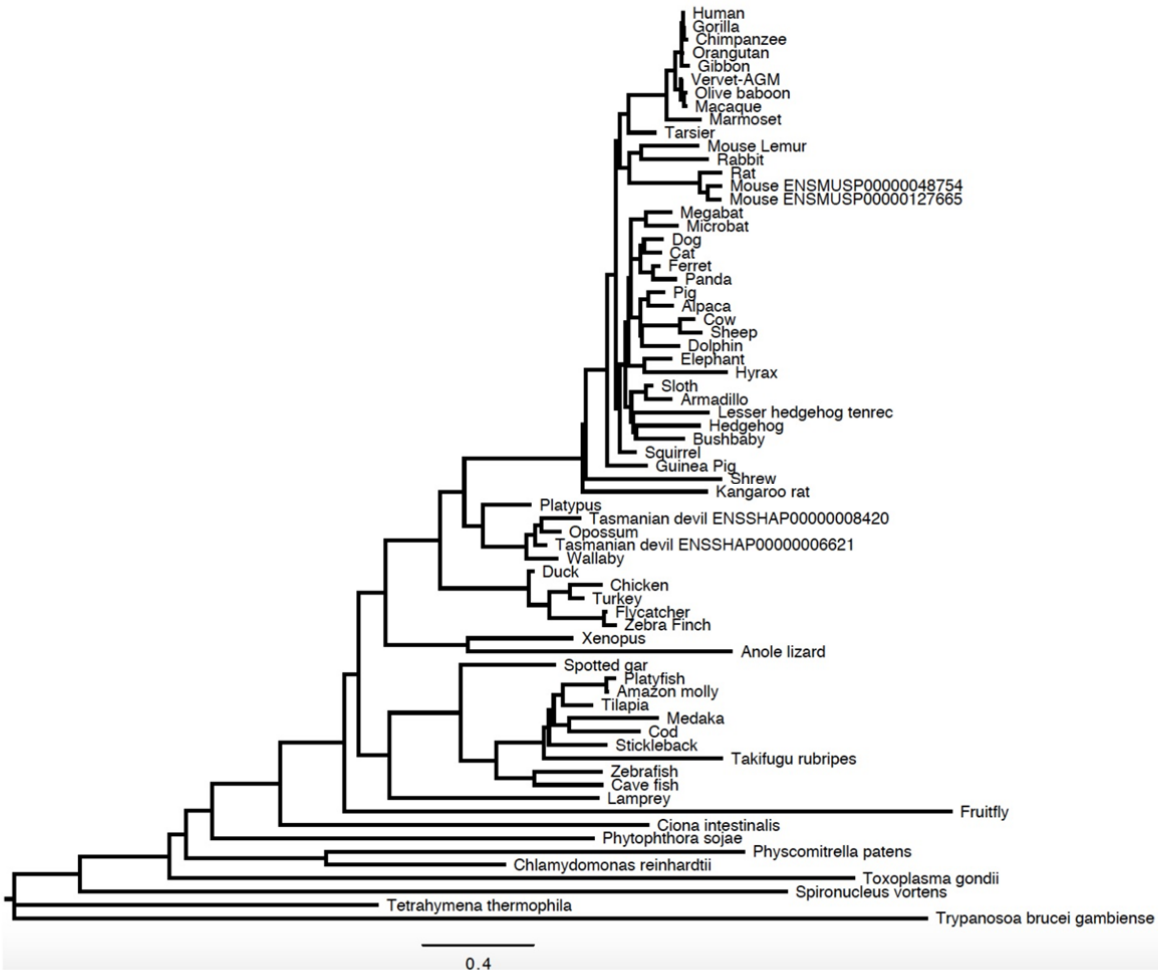

Supplementary Figure 6. Phylogenetic tree modelling of the evolutionary conservation of PIH1D3
Phylogeny of all PIH1D3 orthologs from Ensembl, plus ciliate species of interest. The two mouse homologs of PIH1D3 on chromosome 1 and chromosome X are indicated. Phylogeny generated using PhyML using LG Model and nearest neighbour interchange topology optimisation.[1]

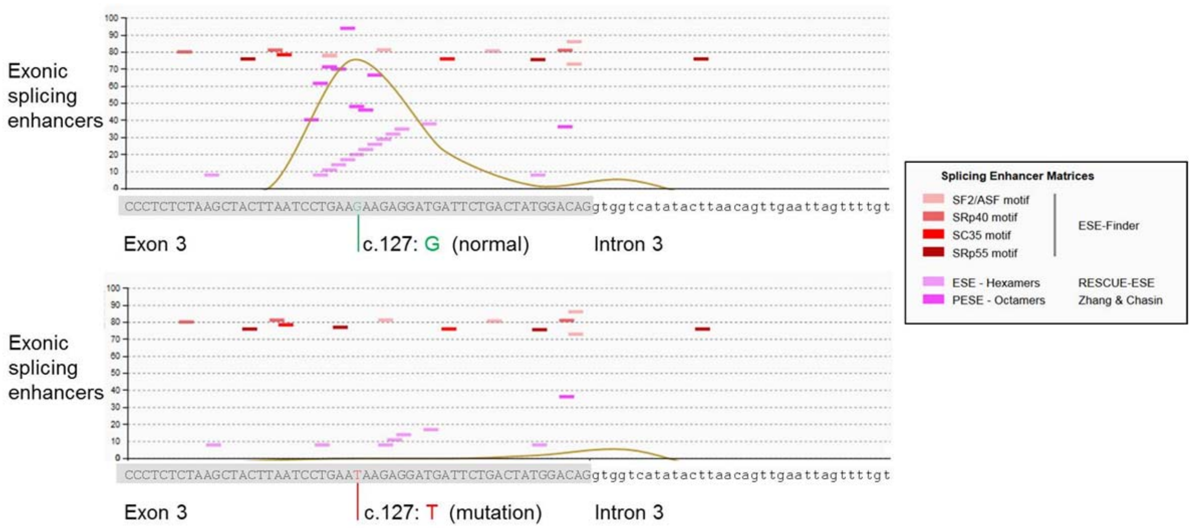

Supplementary Figure 7. (Top) ESE prediction within *PIH1D3* exon 3 by Human Splicing Finder V3.0 tool. (Bottom) The c.127G>T mutation disrupts the 3' ESE of exon 3 (pink). The physiologic intron 3 acceptor splice site is weak (MaxEntScan score: 1.07). An intact exon 3 ESE is, therefore, probably crucial for proper splicing of this exon.

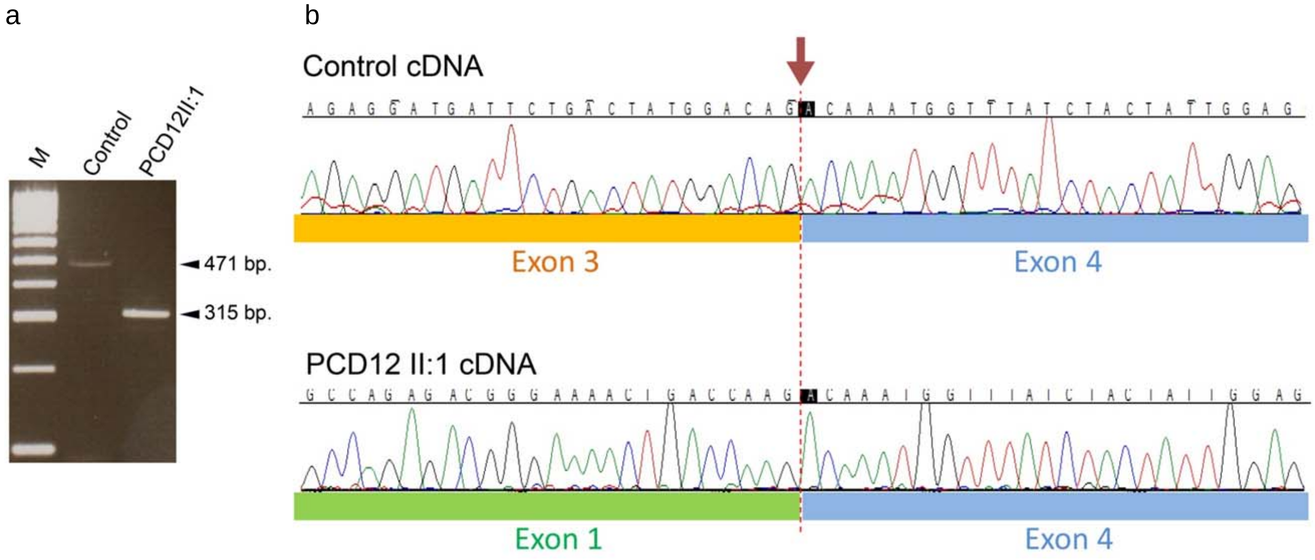

Supplementary Figure 8. RT-PCR of *PIH1D3* transcripts from PCD12 II:1 nasal respiratory epithelial cells
(a) Different sized products are amplified in reverse transcription PCR (RT-PCR) analysis of nasal respiratory epithelial cells of an unaffected control (471 bp) and individual PCD12 II:1 (315 bp) using primers in *PIH1D3* exon 1 and exon 6. The size difference of 156 bp between control and PCD12 II:1 cDNA products corresponds to the length of exon 3. These products were amplified after several attempts to optimize the reaction on limited amounts of patient material. M, molecular marker. (b) Sanger sequencing of the cDNA product amplified in the control showed the expected cDNA splice junction between exon 3 and exon 4 (top). The PCD12 II:1 patient cDNA product showed splicing between exon 1 and exon 4, indicating that exon 3 is absent (bottom). Notably, *PIH1D3* transcripts amplified from respiratory cells in both control and patient did not contain exon 2 (belonging to the 5'UTR of *PIH1D3*), corresponding to NM_173494.1 in Figure 1 of the main paper.

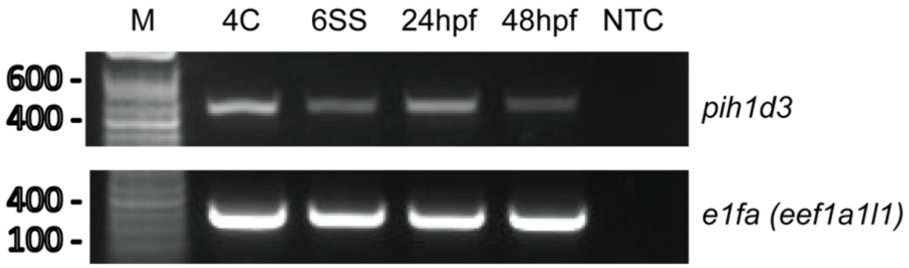

Supplementary Figure 9. Expression of zebrafish *pih1d3* during development
Time course RT-PCR measuring full-length zebrafish *pih1d3* mRNA expression in wild type TuAB embryos. Samples were collected at 4-cell stage (4C), 6-somite stage (6SS, approximately 12 hours post-fertilization (hpf)), 24 hpf and 48 hpf. The non-ciliary *e1fa* mRNA serves as a loading control. NTC, no template control.

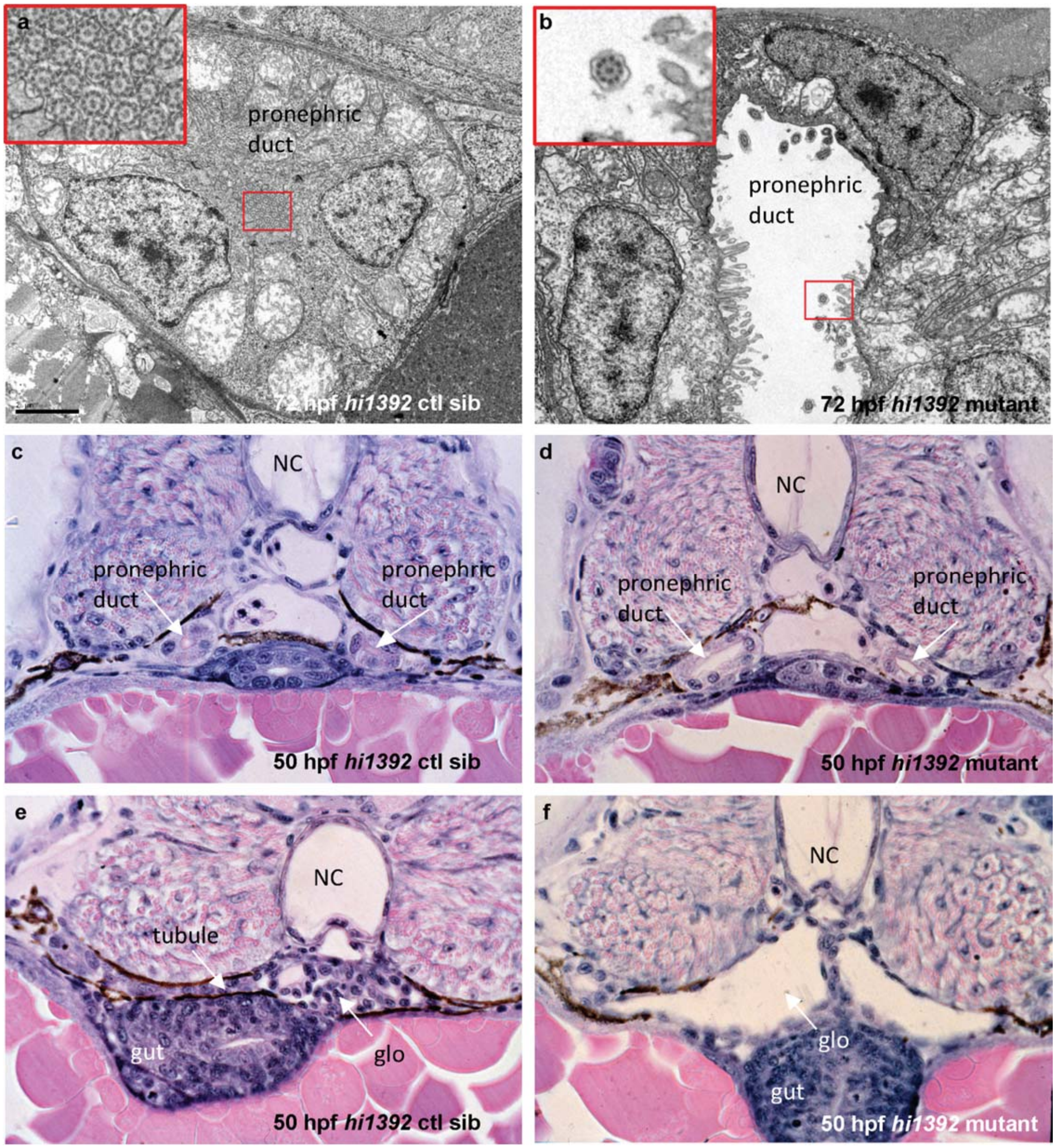

Supplementary Figure 10. Pronephric cyst phenotypes in zebrafish *hi1392Tg* mutants

(a, b) Transmission electron microscopy at 72 hours post fertilization shows dilation of the pronephros in hi1392Tg mutants (b) and disruption of the usual tightly packed arrangement of motile cilia that is seen in controls siblings (ctl sib, a). Scale bar, 2 µm. Insets are higher magnification images of the indicated pronephric cilia. (c-f) Cross sections of hematoxylin and eosin-stained 50 hpf embryos show the dilated pronephric ducts and glomeruli of mutants (d, f) compared to control siblings (c, e). NC, notochord; glo, glomerulus.

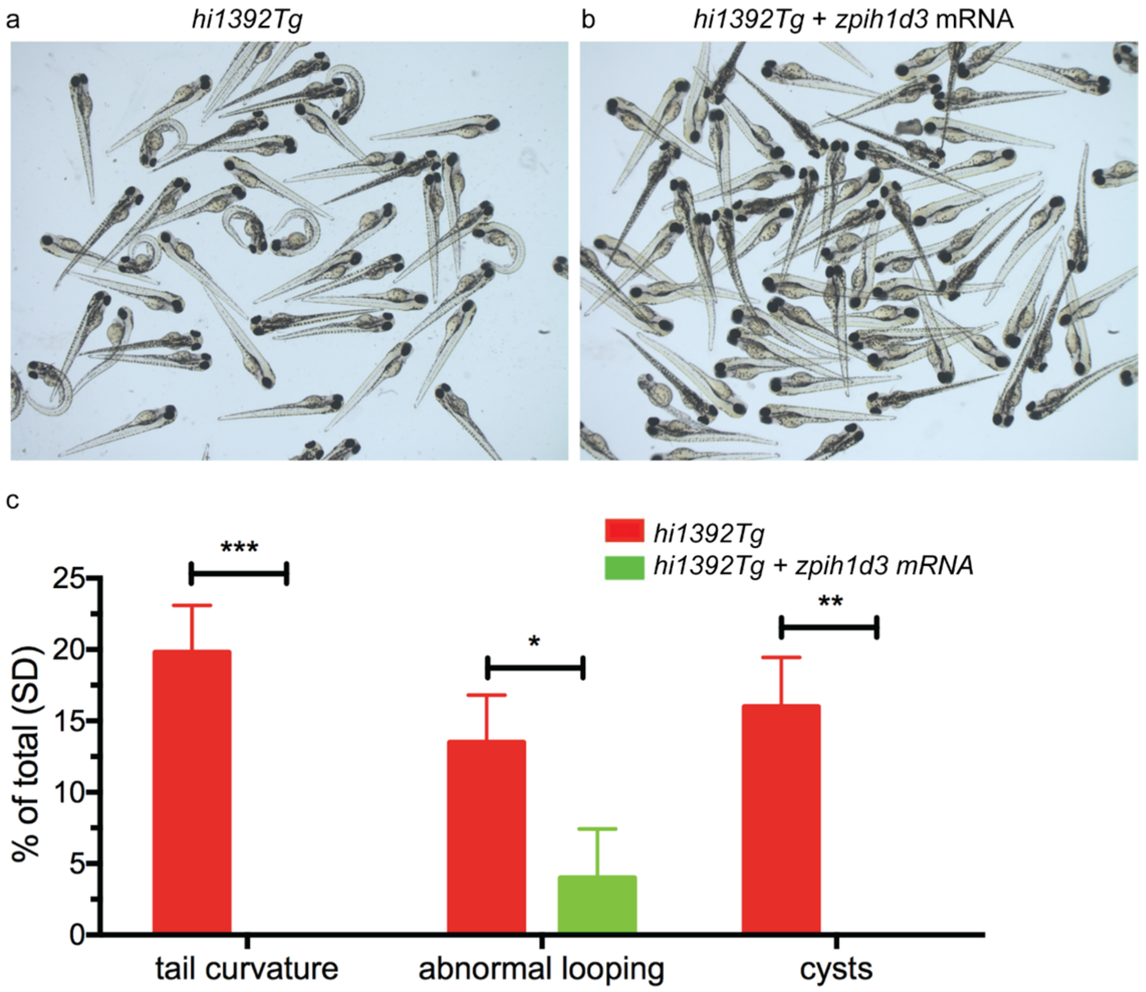

Supplementary Figure 11. Rescue of *hi1392Tg* mutant phenotypes by injection of zebrafish *pih1d3*

(a, b) Representative clutches of 72 hpf *hi1392Tg* mutant embryos injected at one-cell stage with 250 pg of zebrafish *pih1d3* mRNA, showing rescue of mutant phenotypes (b) compared to non-injected siblings (a). (c) Quantification of common phenotypes in 48 hpf *hi1392Tg* mutant embryos (red columns) versus embryos injected with 250pg of zebrafish *pih1d3* mRNA (green columns). There was complete or significant rescue of ventral body axis curvature, pronephric cysts and abnormal cardiac looping (images not shown). Columns represent data from 3 separate experiments with n=29-111 per experiment. Error bars show standard deviation, significance calculated using two way ANOVA, p value *<0.05, **<0.005, ***<0.001.

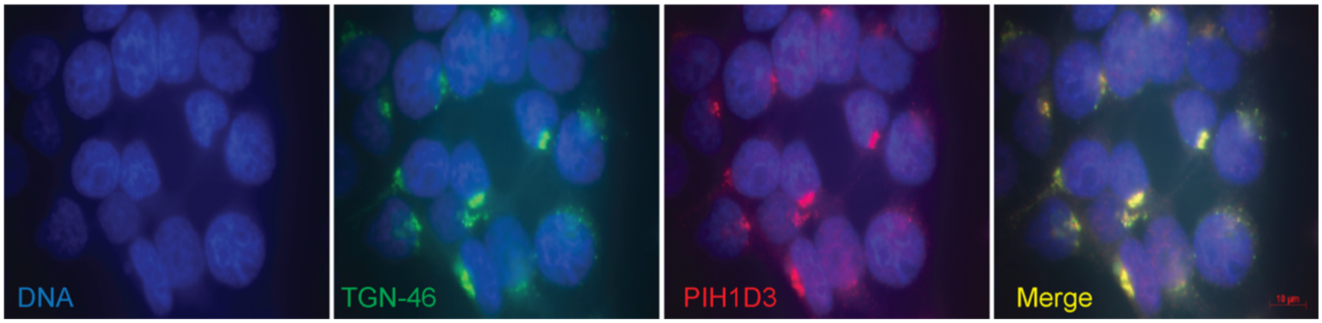

Supplementary Figure 12. Co-localisation of TGN-46 and PIH1D3 in HEK293 cells
HEK293 transfected with GFP-tagged PIH1D3 (anti-GFP in red, Roche) shows co-localisation with endogenous TGN-46 (green, AbD Serotec). Nuclei in blue, DAPI. Scale bar, 10µM.

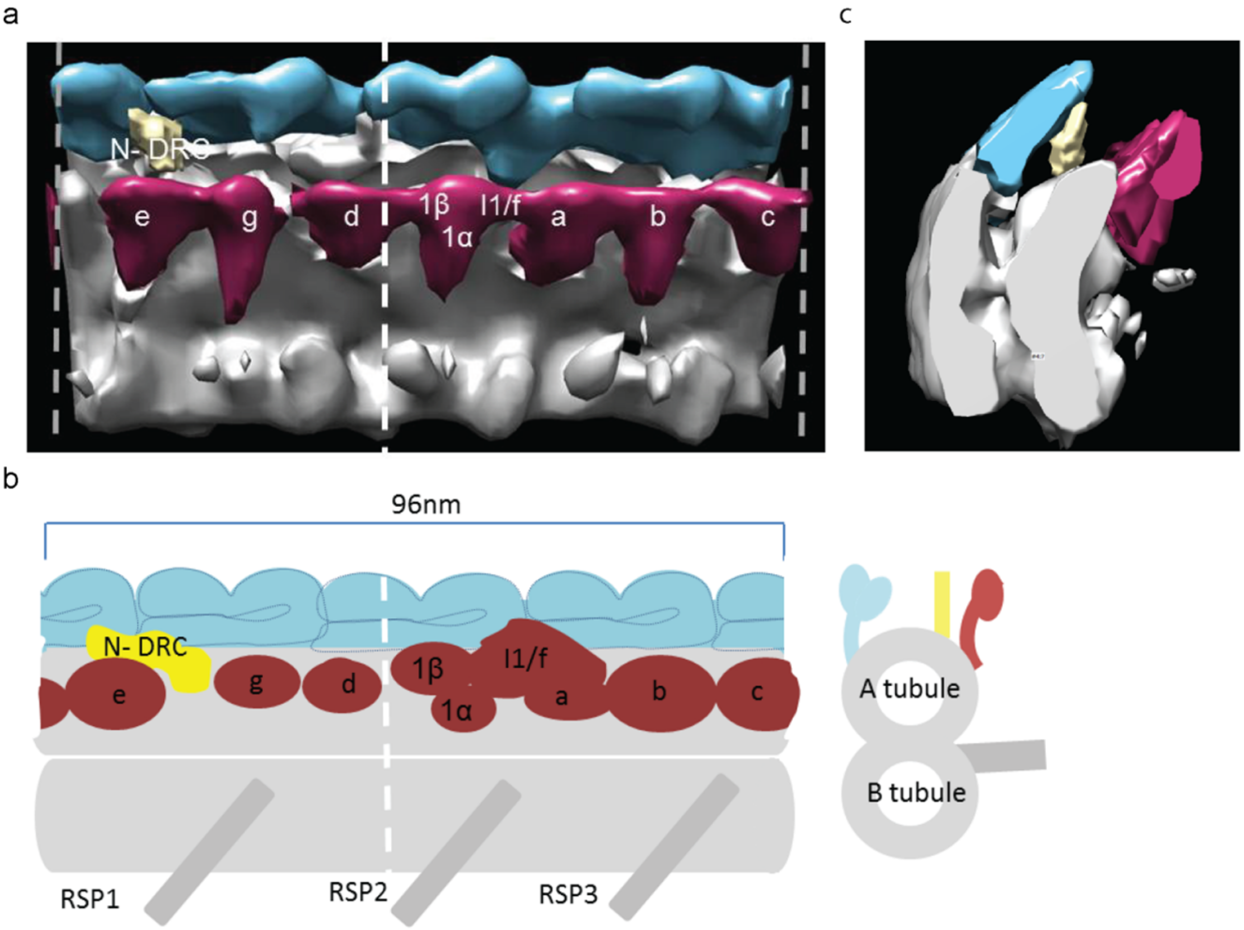

Supplementary Figure 13. Electron tomography of the 96nm repeat including the inner dynein arm of a healthy control
(a) Axonemal structure from an averaged tomogram of the axoneme of a respiratory cilium, constructed from a nasal brushing a healthy male of 31yrs (similar age to PCD12 in main paper Figure 6). The image viewed in longitudinal section was coloured using Chimera software (a) and rotated to 90 degrees to view the microtubule doublet in transverse section (c). (b) A corresponding diagram of the 96nm repeating axoneme in longitudinal (left) and transverse (right) section. Outer dynein arms are depicted in blue, inner dynein arms in red and the dynein regulatory complex in yellow. All components are present in this healthy sample. The dashed white line represents the start of the equivalent diagram and tomogram in Figure 6 of the main paper, grey dashed line indicates the 96 nm span.

a

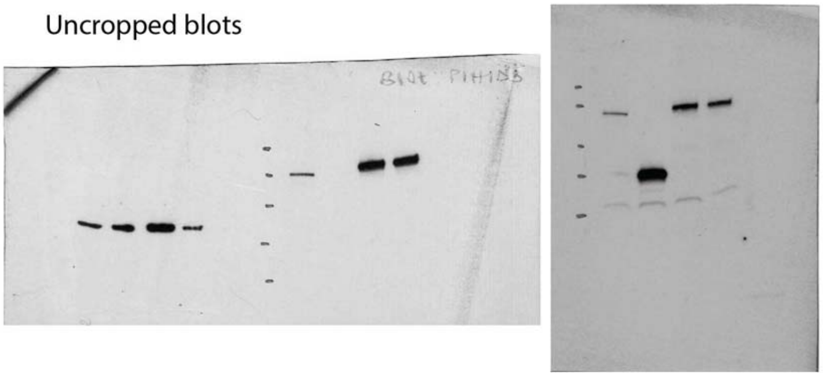

b

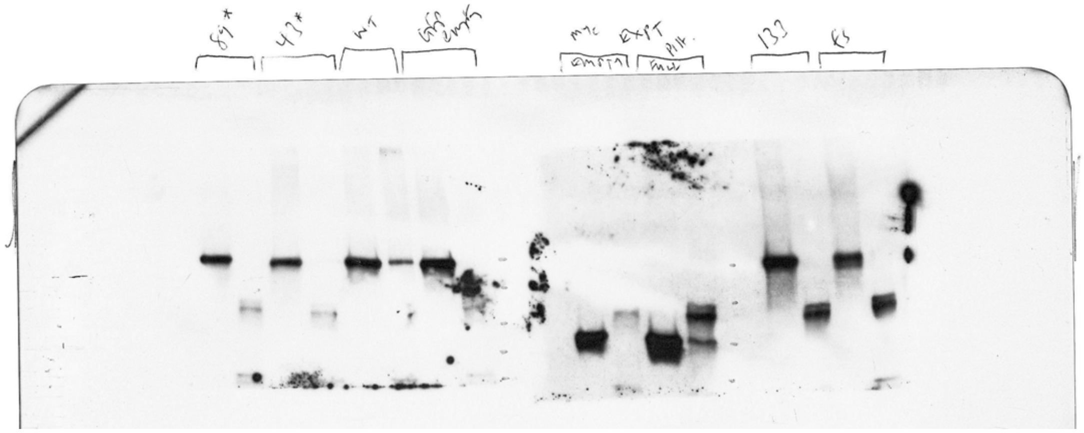

Supplementary Figure 14. Uncropped blots from manuscript
(a) Uncropped blots from Supplementary Figure 3. (b) Uncropped blots from Figure 5, showing data for all of Figure 5c, d. Extra bands cropped from the images are IgG antibody proteins present in the pulldown lanes.

Supplementary Table 1. Coding and non-coding elements within PIH1D3 X chromosome deletions Asterisk indicates only the last 6 coding exons of *IL1RAPL2* are deleted. Indicated in bold are genes referenced in the OMIM database.

	**DCP855**	**DC1337**	**DCP603**	**DCP1747**
**Boundaries of the deletion** (Grch37)	103,077,143-106,814,443	103,580,266-106,853,641	104,582,944-106,510,764	104,582,944-106,510,764
**Deleted genes**	***RAB9B***			
	RNA5SP511			
	ELF2P1			
	LOC644261			
	DPPA3P1			
	TMSB15B			
	H2BFXP			
	H2BFWT			
	H2BFM			
	LOC100101478			
	LOC101060236			
	SLC25A53			
	ZCCHC18			
	LOC286437			
	FAM199X			
	ESX1			
	PHBP10	*PHBP10*		
	RPL18AP14	*RPL18AP14*		
	TEX13A	***TEX13A***		
	IL1RAPL2	***IL1RAPL2***	***IL1RAPL2****	***IL1RAPL2****
	KCTD9P2	*KCTD9P2*	*KCTD9P2*	*KCTD9P2*
	RNU6-207P	*RNU6-207P*	*RNU6-207P*	*RNU6-207P*
	NRK	***NRK***	***NRK***	***NRK***
	SERPINA7	***SERPINA7***	***SERPINA7***	***SERPINA7***
	LOC100131448	*LOC100131448*	*LOC100131448*	*LOC100131448*
	MUM1L1	*MUM1L1*	*MUM1L1*	*MUM1L1*
	LOC107178920	*LOC107178920*	*LOC107178920*	*LOC107178920*
	NAP1L4P2	*NAP1L4P2*	*NAP1L4P2*	*NAP1L4P2*
	SERPINA7P1	*SERPINA7P1*	*SERPINA7P1*	*SERPINA7P1*
	CXorf57	*CXorf57*	*CXorf57*	*CXorf57*
	MIR548AN	*MIR548AN*	*MIR548AN*	*MIR548AN*
	RNF128	***RNF128***	***RNF128***	***RNF128***
	TBC1D8B	*TBC1D8B*	*TBC1D8B*	*TBC1D8B*
	RIPPLY1	***RIPPLY1***	***RIPPLY1***	***RIPPLY1***
	CLDN2	***CLDN2***	***CLDN2***	***CLDN2***
	MORC4	***MORC4***	***MORC4***	***MORC4***
	EEF1A1P40	*EEF1A1P40*	*EEF1A1P40*	*EEF1A1P40*
	RBM41	*RBM41*	*RBM41*	*RBM41*
	NUP62CL	*NUP62CL*	*NUP62CL*	*NUP62CL*
	LOC644563	*LOC644563*	*LOC644563*	*LOC644563*
	PIH1D3	***PIH1D3***	***PIH1D3***	***PIH1D3***
	MYCLP1	***MYCLP1***		
	DNAJA1P3	*DNAJA1P3*		
	KRT18P49	*KRT18P49*		
	FRMPD3-AS1	*FRMPD3-AS1*		
	FRMPD3	*FRMPD3*		

Supplementary Table 2. Clinical features of males carrying *PIH1D3* mutations

WES, whole exome sequencing. NGS, next-generation sequencing. SI, situs inversus. OM, otitis media. NO, nitric oxide. NRD, neonatal respiratory distress. NA, not assessed/available. NR, not relevant. FEV1, lung forced expiratory volume in 1 second measurement. IDA, ODA, inner dynein arm, outer dynein arm.

Individual and Origin	Genetic analysis method	Genotype	Age diagnosed	SI	Bronchiectasis	Respiratory symptoms	Other symptoms	FEV1 (date and age test performed)	Fertility	Otitis media and hearing	Nasal NO	High speed video microscopy	Ultrastructural defect (TEM)
PCD12 II:1 UK	WES	c.127G>T ; p.Glu43*	2 yrs	Yes	Mild bronchiectasis	Recurrent upper and lower respiratory tract infections. Bilateral sinusitis		FEV1 91% predicted (2013 at 27 yrs old)	Complete asthenozoospermia	OM with effusion and hearing difficulties	1999 7.5 nL/min 2009 0.5 nL/min	1989 – Static; 2009 - 60% static, 40% beating with a normal frequency but jittery beat 12.6Hz; 2015 – Mixed beat pattern, some static patches others disorganised beating	1989 (4% total absence, 82% ODA retained); 2009 (33% total absence, 58% ODA retained); 2015 (38% total absence, 45% ODA retained, 2% IDA retained)
PCD392 II:1 Sri Lanka	NGS gene panel	c.266G>A ; p.Trp89*	17 yrs	No	Bronchiectasis (bilateral lower lobes & middle lobe)	Recurrent upper and lower respiratory tract infections. Sinusitis	Asthma, dev delay	FEV1 23% predicted (2015 at 25 yrs old - difficulty with technique)	NR	No hearing issues	2008 40nL/min	2008 Jan – Static 0Hz; 2008 April – Static 0Hz	2008 (14% total absence, 61% IDA retained); 2008 (9% total absence, 82% IDA retained)
DC121 (DCP68) France	Sanger sequencing	c. 397G>T; p.Asp133Tyr	4 yrs	No	Mild bronchiectasis	Recurrent upper and lower respiratory tract infections		FEV1 89% predicted (2016 at 17 yrs old)	NR	OM	NA	2000 Oct - Static 0Hz; 2003 Jul - Static 0Hz	
GVA30 II:1 Spain	WES	c.489_492del; p.Ile164Leufs*11	NA	Yes	Bilateral lower lobe bronchiectasis	NRD. Upper respiratory tract infections since birth. Chronic rhinorrea and cough, recurrent pneumonia from very young age. Sinusitis	Type II narcolepsy	NA	Complete asthenozoospermia	OM with effusion and hearing difficulties	NA	Static (0Hz)	2016 (85% total absence, 6% ODA retained, 9% IDA retained)
GVA30 II:2 Spain	WES	c.489_492del; p.Ile164Leufs*11	NA	Yes	Bilateral lower lobe bronchiectasis	NRD. Upper respiratory tract infections since birth. Chronic rhinorrea and cough, recurrent pneumonia from very young age. Sinusitis	Type II narcolepsy	NA	Complete asthenozoospermia	OM with effusion and hearing difficulties	NA	Static (0Hz)	NA

GVA30 II:3 Spain	WES	c.489_492del; p.Ile164Leufs*11	NA	Yes	Bilateral lower lobe bronchiectasis	NRD. Upper respiratory tract infections since birth. Chronic rhinorrea and cough, recurrent pneumonia from very young age. Sinusitis	Type II narcolepsy	NA	Complete asthenozoospermia	OM with effusion and hearing difficulties	NA	Static (0Hz)	NA
DCP855 (DC518) France	SNP array	3.73-Mb deletion	6 yrs	No	Bronchiectasis	NRD. Recurrent upper and lower respiratory tract infections	Intellectual disability	Unknown	NR	OM	NA	2011 Feb - Static 0Hz (HSV)	IDA+ODA (4 biopsies: very few cilia, no quantification)
DCP1337 (DC864) France	Sanger sequencing, SNP array	3.27-Mb deletion	9 yrs	No	Bronchiectasis (in middle lobe), lobectomy	NRD. Recurrent upper and lower respiratory tract infections	Transient mild cerebellar syndrome, delayed milestones	FEV1 72% predicted (2015 at 12 yrs old)	NR	OM	NA	2012 June - Static 0Hz	IDA+ODA (97% total absence; 3% retained ODA)
DCP603 (DC393) Morocco	SNP array	1.93-Mb deletion	Birth	Yes	Bronchiectasis (in left and right lower lobes and middle lobe)	NRD. Recurrent upper and lower respiratory tract infections		Obstruction and hypoxemia	NR	OM	7 nL/min	2001 Feb - Static 0Hz; 2008 Sep - Static 0Hz	IDA+ODA (100% total absence)
DCP1747 (DC393) Morocco	SNP array	1.93-Mb deletion	3 yrs	No	No bronchiectasis	NRD. Recurrent upper and lower respiratory tract infections		FEV1 96% predicted (2014 at 5 years old)	NR	OM, severe bilateral hearing loss	NA	2012 March - Static 0Hz	IDA+ODA (93.5% total absence; 1.5% retained IDA; 5% retained ODA)
DCP894 (DC539) France	Sanger sequencing	c.263_268delinsG; p.Ile88Argfs*12	29 yrs	No	Bronchiectasis	Recurrent upper and lower respiratory tract infections		Unknown	Asthenozoospermia	OM	7 nL/min	2011 June - Static 0Hz (HSV)	IDA+ODA (97% total absence; 0.5% retained IDA; 2.5% retained ODA)
DCP1218 (DC768) France	Sanger sequencing	c.511C>T; p.Gln171*	Birth	Yes	Mild bronchiectasis (in middle lobe)	NRD. Recurrent upper and lower respiratory tract infections		FEV1 48% predicted (2016 at 13 yrs old)	NR	OM	NA	2011 Sep - Static 0Hz	IDA+ODA (90% total absence; 3% retained IDA; 7% retained ODA)
DCP1849 (DC768) France	Sanger sequencing	c.511C>T; p.Gln171*	Birth	No	No bronchiectasis	NRD. Cough and rhinitis		NA	NR	OM	NA	NA	NA

Supplementary Table 3. Primers used in this study

Gene	Primers
**Gateway**	
PIH1D3	5’-CACCGAATCTGAAAATATGGATTCTGAA-3’
	5’ TCAGAAGAAATTAGCAATATCTAA-3’
DNAI2	5’-CACCGAGATTGTGTACGTGTACGTC-3’
	5’-CTAGGCTAAGTCTTCTTCCACTTC-3’
**SD mutagenesis**	
PIH1D3 (p.E43*)	5’-CTACTTAATCCTGAATAAGAGGATGATTCTG-3’
	5’-CAGAATCATCCTCTTATTCAGGATTAAGTAG-3’
PIH1D3 (p.W89*)	5’GGAAAATAATGAGGACATCTAGAATTCAGAAGAGATTCCAGA-3’
	5’-TCTGGAATCTCTTCTGAATTCTAGATGTCCTCATTATTTTCC-3’
PIH1D3 (p.D133Y)	5’-AGGGTTGTCAAAAAAGTACTCCTCAACAGGTTG-3’
	5’-CAACCTGTTGAGGAGTACTTTTTTGACAACCCT-3’
PIH1D3 (p.T163_I164delfs)	5’-AAATTGATATCCAGGAAACCTTGACCTTCGTACTCCTC-3’<br>5’-GAGGAGTACGAAGGTCAAGGTTTCCTGGATATCAATTT-3’
**RT-qPCR in ciliogenesis**	
PIH1D3	5’-GTTGTTGCAGTGAACTAGTGG-3’
	5’-TTATCAACAGCTTCTTCTGAGGAGT-3’
DNAI2	5’-AAGGAGAAGGGTAAGGCGGA-3’
	5’-GACTTGGTTGCTGAGGCACT-3’
DNAH5	5’-TGCAGATGCCATGGTTCACT-3’
	5’-ATGAAGCCAACCTCGTCAGG-3’
GAPDH	5’-TGCACCACCAACTGCTTAGC-3’
	5’-GGCATGGACTGTGGTCATGAG-3’
**Genomic PCR primers to Sanger sequence PIH1D3 (start at exon 3 because exons 1,2 are non-coding)**	
PIH1D3 exon 3F	5’-TCCAGGTCTGTGAGTTAGCAAAA-3’
PIH1D3 exon 3R	5’-TCATGCATAATCAGCCTTGTAGC-3’
PIH1D3 exon 4F	5’-TCACTGAGCTGGAACTAGGGT-3’
PIH1D3 exon 4R	5’-ACCACACGTGACTTTCTTGGTC-3’
PIH1D3 exon 5F	5’-TGCATGGAAAAGATATCAAAGCGT-3’
PIH1D3 exon 5R	5’-AACTGAAAGGAAGGTGTTATGCTTT-3’
PIH1D3 exon 6F	5’-TATATGCCTGAAGACCTGCAAGC-3’
PIH1D3 exon 6R	5’-GGACGGAACTAGGATGGAAGAAA-3’
PIH1D3 exon 7F	5’-TTTGGAGCCAGAAACCTTAGTCA-3’
PIH1D3 exon 7R	5’-CCACTTTTCTTCAGACTTTAGGGG-3’
PIH1D3 exon 8F	5’-GCCTGTAAACATAGCCTGAGACT-3’
PIH1D3 exon 8R	5’-AAAGCACAACTGAAGAAGCCAAA-3’
**RT-PCR in PCD12**	
PIH1D3 exon 3F	5’-TTACAGCTCTGGAAGCCCT -3’
PIH1D3 exon 5R	5’-TTTCCTCGCTGGTTTCAGGG-3’
PIH1D3 exon 6R	5’-CTTCAGTTCCCACCTGCTGT-3’
**Genomic primers for mutation confirmation and segregation**	
PCD12 II:1 F	5’-ACAGCTCTGGAAGCCCTCTC-3’
PCD12 II:1 R	5’-AGCCTTGTAGCTTCCTGTGA-3’
PCD392 II:1F	5’-TGAACCCAAACTGAGTGTGAAGA-3’
PCD392 II:1R	5’-CAATTACACATGCCTTCCGTGT-3’
DCP68F	5’-TATATGCCTGAAGACCTGCAAGC-3’
DCP68R	5’-GGACGGAACTAGGATGGAAGAAA3’
GVA II:1 30F	5’- TGGAGCCAGAAACCTTAGTCA-3’
GVA II:1 30R	5’- ACCTTACAGCCCCACAAAATG-3’
DCP894F	5’-TGCATGGAAAAGATATCAAAGCGT-3’
DCP894F	5’-AACTGAAAGGAAGGTGTTATGCTTT-3’

DCP1218/1849F	5'-TTTGGAGCCAGAAACCTTAGTCA-3'
DCP1218/1849F	5'-CCACTTTTCTTCAGACTTTAGGGG-3'
DCP855F	NA* (Large multi-gene deletion including *PIH1D3*)
DCP855F	NA* (Large multi-gene deletion including *PIH1D3*)
DCP1337F	NA* (Large multi-gene deletion including *PIH1D3*)
DCP1337F	NA* (Large multi-gene deletion including *PIH1D3*)
DCP603/1747F	NA* (Large multi-gene deletion including *PIH1D3*)
DCP603/1747F	NA* (Large multi-gene deletion including *PIH1D3*)

*: not applicable, large multigene deletion characterised using SNP arrays.

Supplementary Table 4. Primary antibodies used in this study

Antigen	Antibody	Host Species	Source	Application
GM130	35/GM130	IgG1, Mouse	BD Transduction	IF (1:100)
TGN46	AHP 500	IgG, Sheep	AbD Serotec	IF (1:500)
GFP	7.1 and 13.1	IgG1, Mouse	Roche	co-IP (1µg), WB (1:1000-1500)
MYC	9E10	Mouse	Merck Millipore	WB (1:3000)
GAPDH	6C5	Mouse	Merck Millipore	WB (1:500)

Supplementary Table 5. Secondary antibodies used in this study

Antigen	Host Species	Source	Application
Alexa 488, 594 – conjugated anti rabbit IgG (H+L)	Goat	Invitrogen (Molecular Probes)	IF (1:1000)
Alexa 488, 594 – conjugated anti mouse IgG1	Goat	Invitrogen (Molecular Probes)	IF (1:1000)
Alexa 488, 594 - conjugated anti mouse IgG2b	Goat	Invitrogen (Molecular Probes)	IF (1:1000)
Alexa 488 – conjugated anti mouse IgG (H+L)	Donkey	Invitrogen (Molecular Probes)	IF (1:1000)
Alexa 488 – conjugated anti sheep IgG (H+L)	Donkey	Invitrogen (Molecular Probes)	IF (1:1000)
ECL α-Mouse IgG, HRP-conjugated	Sheep	GE Healthcare UK Ltd	WB (1:3000)
ECL α-Rabbit IgG, HRP-conjugated	Donkey	GE Healthcare UK Ltd	WB (1:3000)

Supplementary references

1. Guindon, S., Lethiec, F., Duroux, P. & Gascuel, O. PHYML Online--a web server for fast maximum likelihood-based phylogenetic inference. *Nucleic Acids Res* **33**, W557-9 (2005).
2. Di Tommaso, P. *et al.* T-Coffee: a web server for the multiple sequence alignment of protein and RNA sequences using structural information and homology extension. *Nucleic Acids Res* **39**, W13-7 (2011).
3. Robert, X. & Gouet, P. Deciphering key features in protein structures with the new ENDscript server. *Nucleic Acids Res* **42**, W320-4 (2014).
4. Kelley, L.A., Mezulis, S., Yates, C.M., Wass, M.N. & Sternberg, M.J. The Phyre2 web portal for protein modeling, prediction and analysis. *Nat Protoc* **10**, 845-58 (2015).
